# Applications of catalytic systems containing DNA nucleobases (adenine, cytosine, guanine, and thymine) in organic reactions

**DOI:** 10.1039/d4ra07996e

**Published:** 2025-01-31

**Authors:** Zahra Khademi, Kobra Nikoofar

**Affiliations:** a Department of Organic Chemistry, Faculty of Chemistry, Alzahra University P.O. Box 1993891176 Tehran Iran k.nikoofar@alzahra.ac.ir kobranikoofar@yahoo.com +982188041344 +982188041344

## Abstract

In recent years, nucleobases have attracted special attention because of their abundant resources and multiple interaction sites, which enable them to interact with and functionalize other molecules. This review focuses on the catalytic activities of each of the four main nucleobases found in deoxyribonucleic acid (DNA) in various organic reactions. Based on the studies, most of the nucleobases act as heterogeneous catalytic systems. The authors hope their assessment will help chemists and biochemists to propose new procedures for utilizing nucleobases as catalysts in various organic synthetic transformations. The review covers the corresponding literature published till the end of August 2023.

## Abstract 2

In recent years, nucleobases have attracted special attention because of their abundant resources and multiple interaction sites, which enable them to interact with and functionalize other molecules.

## Graphical abstract

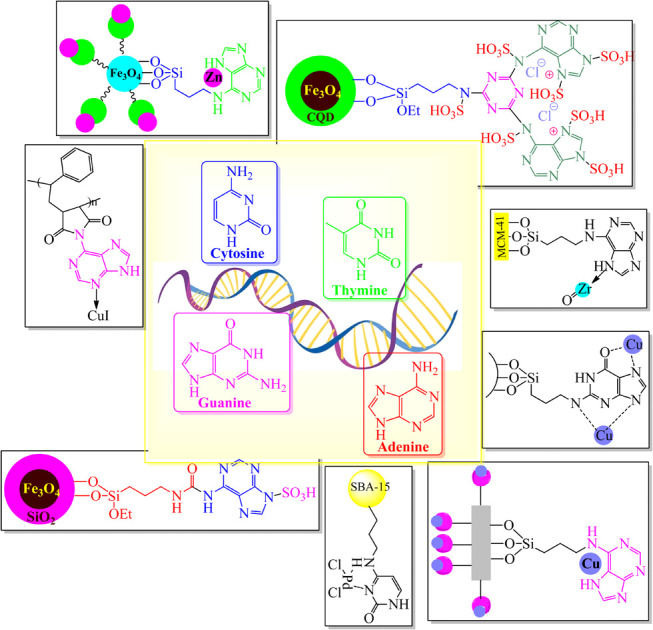

## 1. Introduction

**Scheme 1:**
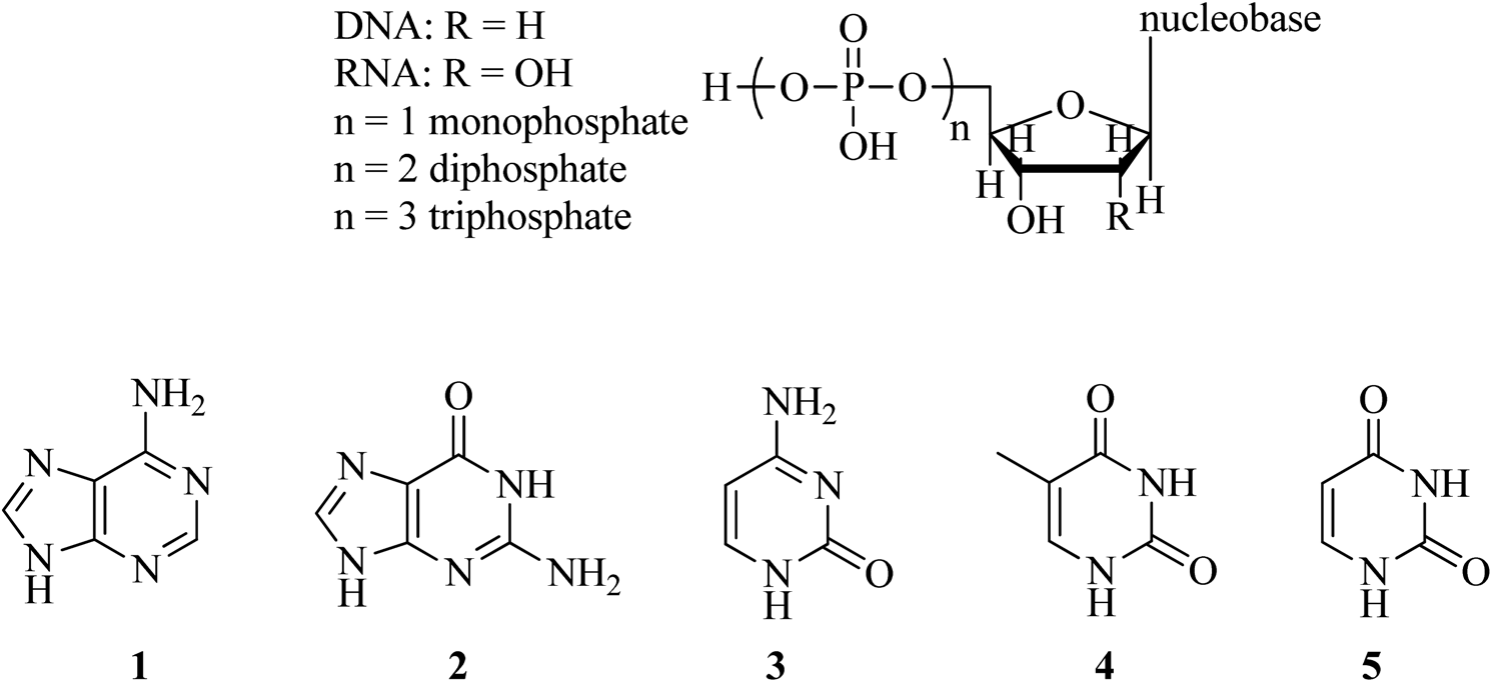
Structures of nucleobases, nucleosides, and nucleotides.

Nucleobases, such as adenine (A, 1), guanine (G, 2), cytosine (C, 3), thymine (T, 4), and uracil (U, 5), are the fundamental units of the genetic code and have been found in DNA (Scheme 1). Nucleosides are made up of a sugar ring (2′-deoxyribose for DNA and ribose for RNA) and a nucleobase. Nucleotides contain a nucleoside and at least one phosphate unit.

The bases A, T, C, and G are found in deoxyribonucleic acid (DNA), while A, U, C, and G are present in RNA. It is widely known that A binds to T (or U), while G pairs with C through hydrogen bonding. Due to the capability of nucleobases to form base pairs and stack upon one another, they are capable of forming long-chain helical frameworks, such as those found in DNA and RNA.^1–4^ Overall, the nucleobases can interact with each other as well as with other organic/inorganic small molecules.^1^

Adenine and guanine have a fused-ring structure derived from purine; thus, they are classified as purine bases. On the other hand, cytosine, uracil, and thymine consist of a simple heterocyclic aromatic ring derived from pyrimidine, and hence, they are called pyrimidine bases.^5,6^

Adenine was first synthesized as a white powder by Oro in 1960.^7^ This compound was produced by heating concentrated ammonium cyanide (NH_4_CN, 1–15 M) at 27 °C to 100 °C for several days, followed by the elimination of a black polymer through centrifugation and the reaction of the supernatant with HCl.^8^

Thymine was first isolated by Kossel and Neumann in 1893 from calf thymus glands, followed by its first synthesis through hydrolysis of the related nucleoside derived from natural sources. In the early 1900s, Fischer presented a synthetic procedure starting from urea, but a more applicable method utilized methylisothiourea instead of urea in the condensation reaction with ethyl formyl propionate in water to form the pyrimidine intermediate, which was then subjected to acidic hydrolysis to afford thymine, with methanethiol as a by-product.^9^

In 1984, cytosine was isolated through the hydrolysis of calf thymus tissue. Its structure was identified in 1903, and it was first synthesized from 2-ethylthiopyrimidin-4(3*H*)-one.^10^

The first isolation of guanine was reported in 1846 by Unger from the excreta of sea birds as a mineral. Subsequently, between 1882 and 1906, the guanine structure was discovered, and the transformation of uric acid into guanine was revealed.^11,12^

Uracil was originally identified in 1900 by Ascoli and then isolated *via* hydrolysis of yeast nuclein.^13^ This compound was also detected in bovine thymus and spleen.^14^

**Scheme 2:**
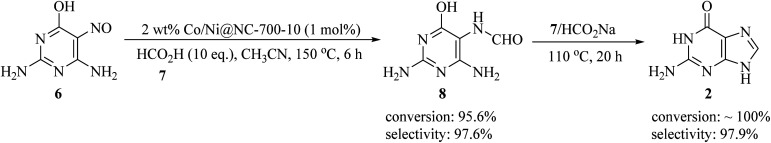
Synthetic procedure for guanine form DANHP and formic acid.

Because of the widespread utility of nucleobases, many studies have been published on different synthesis methods for nucleobases. For example, in 2022, Wang *et al.* prepared a nitrogen-doped carbon-based Co/Ni bimetallic catalyst (2 wt% Co/Ni@NC-700-10) using chitosan as the nitrogen and carbon source. It was found to be effective for guanine formation, utilizing 2,4-diamino-5-nitroso-6-hydroxypyrimidine (DANHP, 6) as starting material. In the first step, the reaction was initiated *via* the one-pot reductive *N*-formylation of 6 with formic acid (7) using 2 wt% Co/Ni@NC-700-10 in acetonitrile media at 150 °C to obtain 2,4-diamino-5-formyl-6-hydroxypyrimidine (DAFHP, 8) with excellent conversion (95.6%) and selectivity (97.6%), which after isolation underwent a cyclization reaction in the presence of formic acid (7) and sodium formate at 110 °C to achieve guanine (2). Based on the resulting HPLC spectra, the conversion of 8 was about 100% with an excellent selectivity of 97.9% (Scheme 2).^15^

**Scheme 3:**
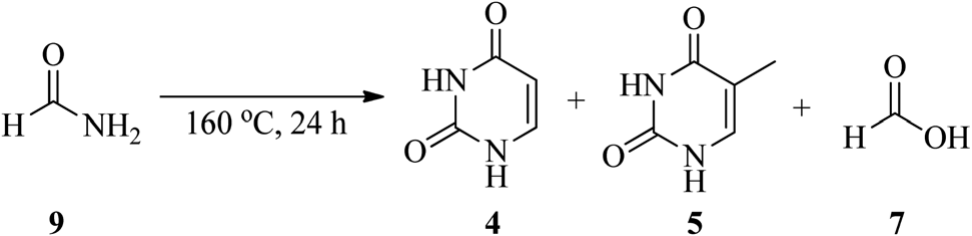
Synthetic procedure for thymine.

**Scheme 4:**
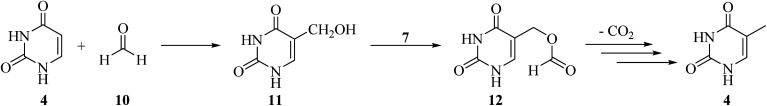
Mechanism for the conversion of uracil to thymine.

In 2021, an article entitled “Prebiotic route to thymine from formamide-a combined experimental–theoretical study” focused on the catalyst-free conversion of uracil to thymine through the thermolysis of formamide (9) at 160 °C within 24 h. This reaction progressed in the presence of formic acid (7) as a key molecule formed *via* the hydrolysis of 9 (Scheme 3). It is important that the disproportionation of formic acid (7) resulted in the formation of CO_2_ and formaldehyde (10), and the latter compound plays an essential role in the hydroxymethylation of uracil^16^ in the first step of the transformation procedure. The reaction was followed by the esterification of the hydroxyl group of 5-hydroxymethyluracil (11) to give 12, which finally rearranged to thymine (4) upon the removal of CO_2_ (Scheme 4).^17,18^

In 2020, Yadav *et al.* reported a review article entitled “Chemistry of abiotic nucleotide synthesis”, which pointed to the prebiotic synthesis of nucleobases from HCN derivatives, such as formamide and urea^19^ or by other sources, with the exception of HCN and formamide.^20^ In 2018, a review entitled “Origins of building blocks of life: a review” reported various methods for the synthesis of nucleobases under simulated prebiotic conditions.^21^

In addition to the above-mentioned methods, the nucleobases could be generated under various conditions. For example, the Nelson group in 2001 studied the synthesis of cytosine and uracil from urea and cyanoacetaldehyde at 100 °C under dry-down conditions and in solution at 4 °C and −20 °C.^22^ In 2004, Orgel prepared adenine *via* the photochemical conversion of the tetramer of hydrogen cyanide in eutectic solution to 4-amino-5-cyano-imidazole.^23^ Cleaves *et al.* in 2006 reported a simple prebiotic procedure to obtain cytosine and uracil upon freezing in solution.^24^ Menor-Salvan *et al.* in 2009 synthesized cytosine and uracil from cyanoacetylene/cyanoacetaldehyde and frozen urea solution under a methane/nitrogen atmosphere.^25^ In 2007, they also obtained some purine bases through the spark activation of an atmosphere of methane, nitrogen and hydrogen in the presence of an aqueous aerosol.^26^

Formamide (which could be obtained from hydrogen cyanide and water) has received potential pre-genetic and pre-metabolic interest and is sometimes considered the origin of life.^27^

Owing to the critical role of formamide in the prebiotic synthesis of nucleobases, numerous studies have been performed utilizing formamide as a possible source of nucleobases under different experimental conditions.^28^ Barks *et al.* in 2010 reported the catalyst-free synthesis of adenine and guanine from UV-irradiated formamide solutions.^29^ In addition, several mineral and metal oxide catalysts were utilized to obtain various DNA-nucleobases from formamide. Saladino *et al.* in 2001 reported the catalytic (in the presence of CaCO_3,_ silica, alumine, kaolin, and zeolite-Y) formation from neat formamide.^30^ Some other catalytic systems to obtain DNA-nucleobases from the formamide substrate are: TiO_2_,^31^ montmorillonites,^32^ cosmic-dust analogues,^33^ iron–sulfur and iron–copper–sulfur minerals,^34^ zirconia,^35^ common rock-forming minerals (such as silicates, and carbonates),^36^ and iron oxides/hydroxides (hematite, goethite, and akaganeite).^37^ In 2022, Nejdl *et al.* utilized ZnCd QDs upon UV irradiation in prebiotic liquid formamide to obtain some nucleobases with increased yields.^38^ Oba *et al.* in 2019 achieved DNA-nucleobases (cytosine, uracil, thymine, and adenine) in interstellar ice analogues composed of simple molecules, including H_2_O, CO, NH_3_, and CH_3_OH after exposure to ultraviolet photons, followed by thermal processes.^39^ In 2013, the preparation of adenine form formamide was considered a self-catalytic mechanism in an abiotic approach.^40^

A considerable number of enzymes consist of nucleobase motifs.^41^ As some enzymes or enzyme complexes require several cofactors, they utilize nucleotide cofactors, including a purine base (usually adenine) binding site.^42^ Adenine is an inextricable part of enzyme cofactors and second messenger systems, such as NAD^+^, FADH2, and cAMP, which are essential for certain catalytic reactions and biochemical processes. In addition, a crucial catalytic role of the adenine moiety is also observed in group II intron catalysis and at the ribosomal peptidyltransferase center.^43^

Nucleobases and their derivatives reveal widespread biological and therapeutic activities in medicinal chemistry as notable pharmacophores,^44,45^ antituberculosis agents,^46^ kinase or cyclin-dependent kinase inhibitors,^47,48^ antibiotics and biofilm inhibitors,^49^ HIV viral capsid inhibitor,^50^ antiviral (such as Human Immunodeficiency Virus and Hepatitis Virus),^51–53^ antineoplastic agents,^54^ and A3 adenosine receptor antagonists and ligands.^55^

Nucleobases possess a wide range of applications in different branches of science and technology (with various roles such as organic/bio-catalyst, reagent, substrate, ligand, capping agent, and promoter) such as polymer preparation and/or modifications,^56–58^ medicinal chemistry and therapeutic investigations,^59^*in vitro* and/or *in vivo* drug delivery^60,61^ nanomaterials and nanotechnology,^62–65^ electrochemistry and electrochemical sensors,^66–70^ supramolecular chemistry,^71–73^ metal–organic frameworks (MOFs) chemistry,^74–76^ heterogeneous catalysts and/or catalytic systems,^77–80^ batteries and energy-storage,^81–83^ oxidation/reduction chemistry,^84–87^ and biodiesel synthesis.^88^ In 1999, some Cu(adenine)_2_ complexes displayed the promotion of O_2_ production from H_2_O_2_ through the disproportionation of hydrogen peroxide into oxygen and water.^89^ Adenine-functionalized conjugated polymer PF6A-DBTO2 demonstrated high photocatalytic activity with hydrogen evolution from water.^90^

The plentiful resources of nucleobases and numerous interaction sites, for instance, hydrogen bonding, π–π stacking, and van der Waals forces, enable them to interact and functionalize other molecules.^91^ Based to these properties, the authors have reviewed the literature reports about the catalytic activity of nucleobases in various organic transformations. It must be mentioned that the number of reports for applications of adenine, cytosine, guanine, and thymine as a sole catalyst or part of the catalytic systems is few, so the catalytic role of each of the four DNA nucleobases id investigated in each part as below.

## 2. Catalytic activity of adenine in various organic transformations

**Scheme 5:**
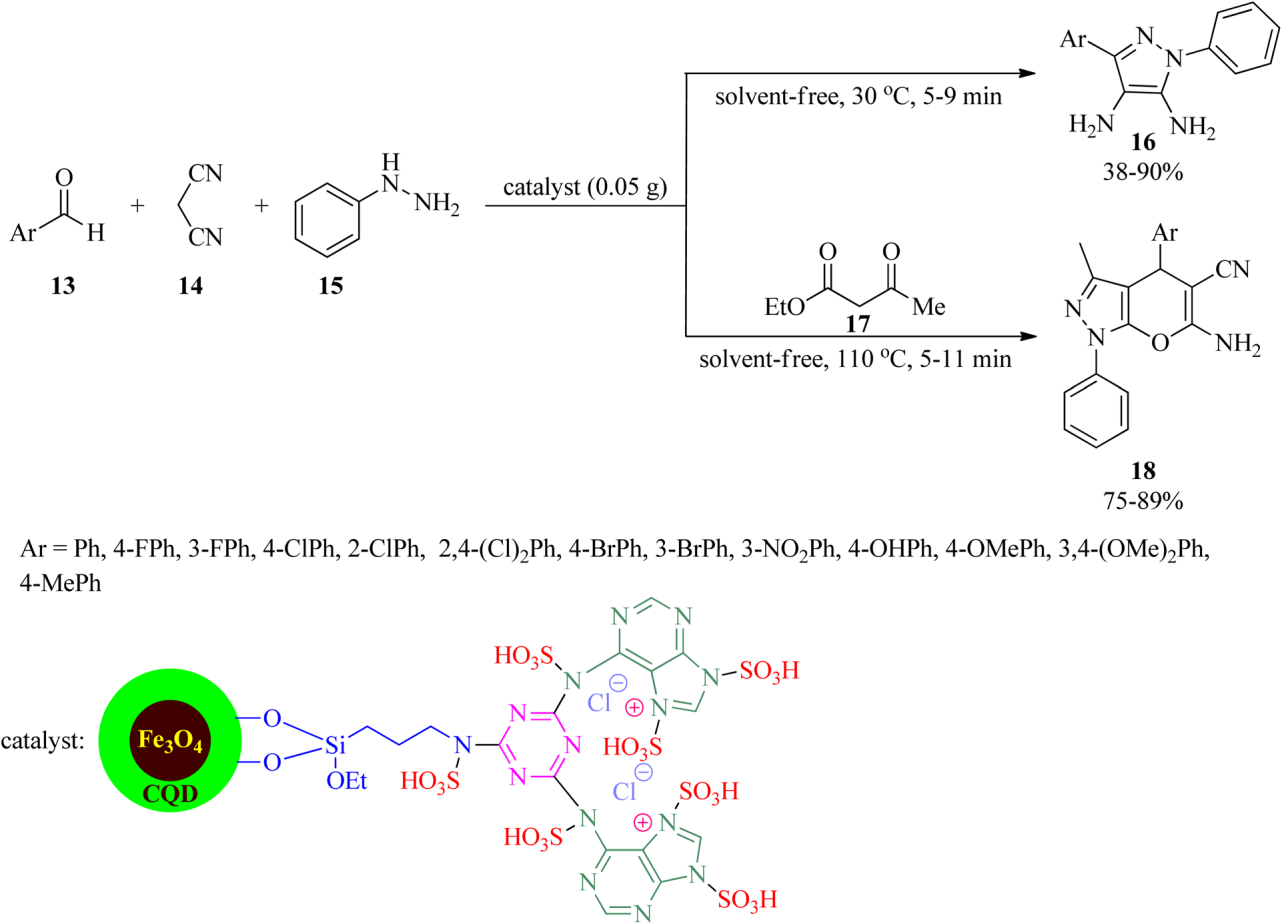
Synthesis procedure for 1*H*-pyrazole 4-carbonitrile and pyrano[2,3-*c*] pyrazoles.

Owing to the key role of pyrazole motif in various fields of chemistry and biology,^92–95^ Ahmadi *et al.* in 2023 presented a novel, applicable, and efficacious technique to prepare 5-amino-1,3-diphenyl-1*H*-pyrazole 4-carbonitrile and pyrano[2,3-*c*]pyrazoles utilizing [Fe_3_O_4_@CQD@Si(OEt)(CH_2_)_3_NH@CC@A@SO_3_H]^+^Cl^−^ catalyst under solvent-free conditions. Firstly, the catalyst was synthesized *via* coating the Fe_3_O_4_ magnetic nanoparticles with carbon quantum dots (CQD), followed by surface modification with (3-propylamine)-triethoxysilane, functionalization by cyanuric chloride (CC) and adenine, and subsequent functionalization with chlorosulfonic acid. Then, 5-amino-1,3-diphenyl-1*H*-pyrazole 4-carbonitrile (16) was obtained *via* the condensation of equimolar amounts of aromatic aldehydes (13) with malononitrile (14) and phenyl hydrazine (15) at 30 °C in low to excellent yields (38–90%) and very short reaction times (5–9 min). On the other hand, the one-pot four-component condensation reaction of 13, 14, 15, and ethyl acetoacetate (17) realized pyrano[2,3-*c*]pyrazoles (18) within short reaction times in good yields (Scheme 5). The magnetic separability and recoverability of the catalyst were examined within 5 runs without notable activity loss.^96^

**Scheme 6:**
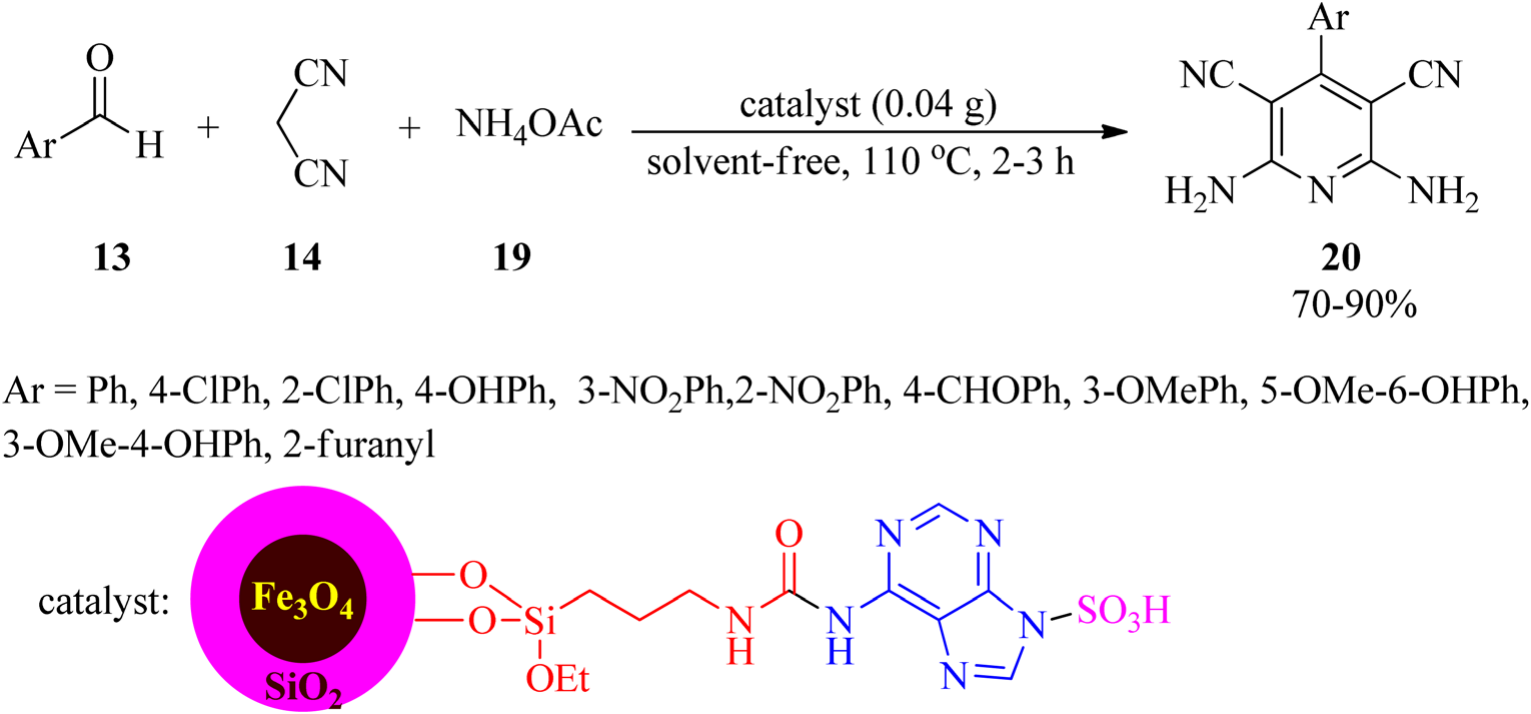
Synthesis of 2,6-diamino-4-arylpyridine-3,5-dicarbonitriles.

In 2022, Sadri *et al.* established a straightforward, convenient, and useful method for the production of 2,6-diamino-4-arylpyridine-3,5-dicarbonitrile (20) *via* the one-pot pseudo-four-component condensation reaction of aldehydes (13) with 14, and ammonium acetate (19) utilizing a nanomagnetic catalyst coated with adenine and sulfonic acid (Fe_3_O_4_@SiO_2_@(CH_2_)_3_NHCO-adenine sulfonic acid), as a facile, cost-effective, recyclable, and reusable catalyst, under solvent-free conditions at 110 °C (Scheme 6). Mild reaction conditions, easy catalyst preparation, simple purification and isolation of the products (no chromatographical techniques) are some of the noteworthy features of this process.^97^

**Scheme 7:**
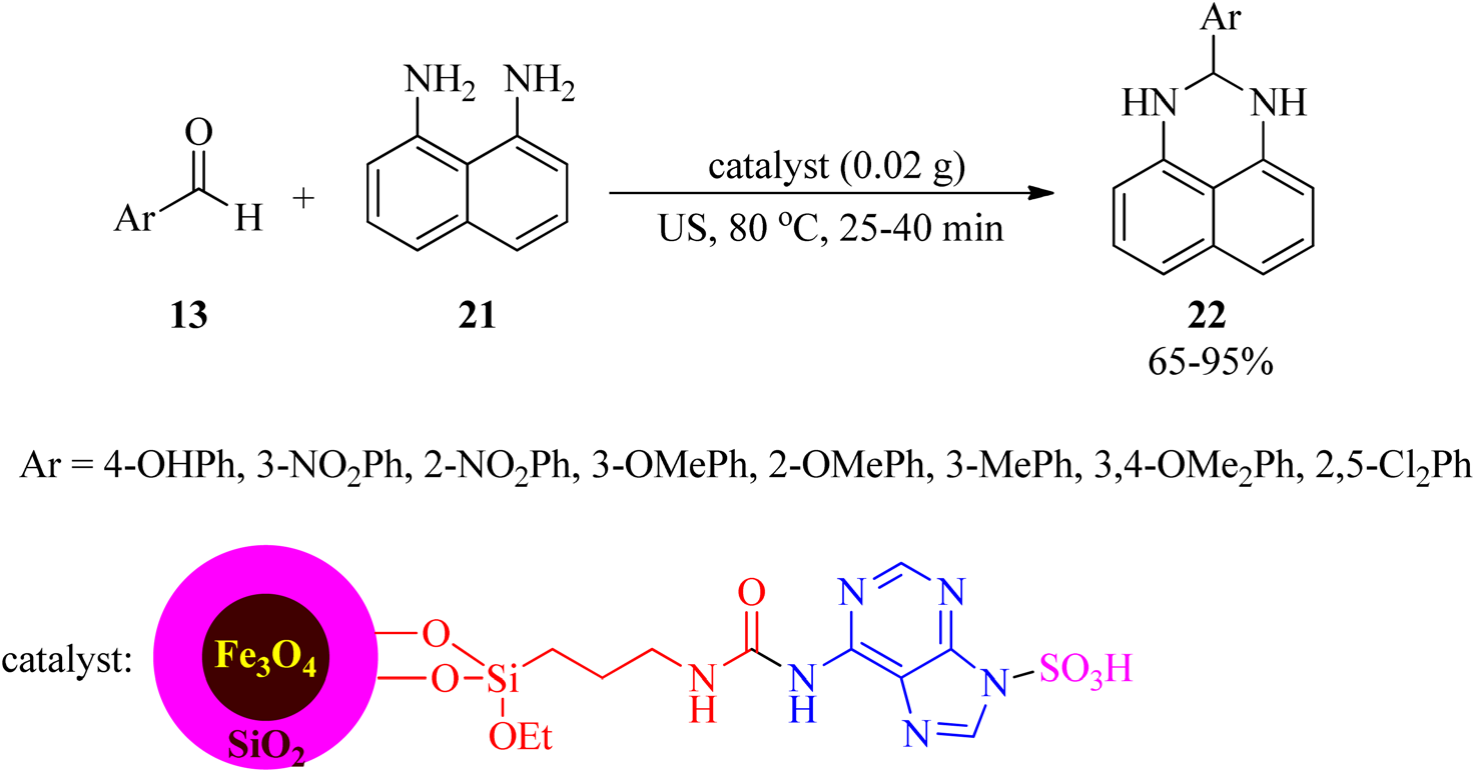
Preparation of 2,3-dihydro-2-aryl-1*H*-perimidines.

The same research group also subjected this catalyst for the synthesis of 2,3-dihydro-2-aryl-1*H*-perimidines (22) using aryl aldehydes (13) and 1,8-diamino naphthalene (21) under ultrasonic irradiation and solvent-free conditions (Scheme 7).^98^ The recovery and reusability test of the nanostructure was successful within 4 runs.

**Scheme 8:**
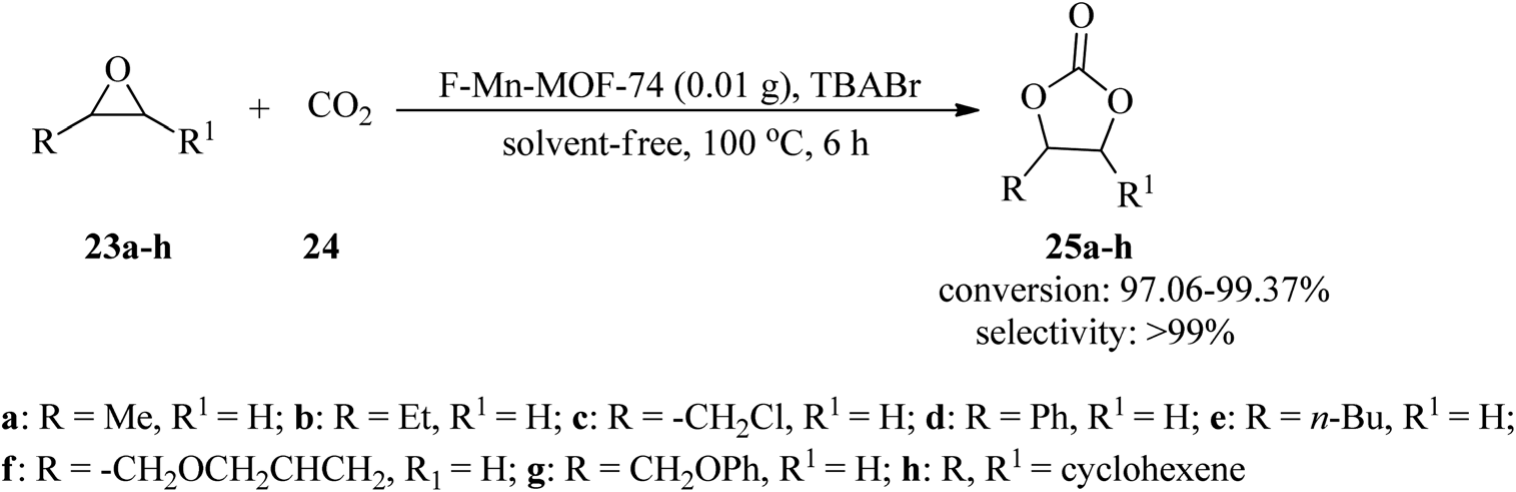
Synthesis of cyclic unsymmetrical carbonates.

In 2020, a highly effective, applicable, and green technique was presented for the formation of cyclic carbonates (25a–h) through the cycloaddition reaction of epoxides (23a–h) with carbon dioxide (24, 10 bar pressure) by flower-like manganeses confined metal–organic framework (F-Mn-MOF-74) as a catalyst and tetrabutylammonium bromide (TBABr) as a co-catalyst under solvent-free conditions at 100 °C within 6 h with excellent conversion and selectivity. In this research, in order to prepare F-Mn-MOF-74, adenine was applied as an alkali source and competitive ligand in comparison to the synthetic Mn-MOF-74. It was found that the spherical F-Mn-MOF-74 catalyst, along with TBABr, disclosed excellent catalytic performance in the cyclic esterification reaction. It should be noted that the catalytic system proceeded with the transformation of cyclohexene oxide with CO_2_ (1 MP) at 160 °C within 24 h to produce the corresponding cyclic carbonate in moderate conversion (51.98%) and excellent selectivity (92.65%) (Scheme 8).^99^ Similarly, this transformation occurred at 80 °C in 8 h utilizing two new adenine-based Zn-(II)/Cd(ii)-MOFs, namely, [Zn_2_(H_2_O)(stdb)_2_(5*H*-A)(9*H*-A)2]_*n*_ (PNU-21) and [Cd_2_(Hstdb)(stdb)(8*H*-A)(A)]_*n*_ (PNU-22), including auxiliary dicarboxylate ligand (stdb = 4,4′-stilbenedicarboxylate). The catalysts were characterized through different techniques, such as single-crystal X-ray diffraction (SXRD), which disclosed 2D and 3D rigid and robust building blocks for PNU-21 and PNU-22, respectively, along with coordinately unsaturated metal surroundings. It should be mentioned that the presence of unsaturated Zn and Cd metals and basic N atoms in both catalysts converted them into acid–base efficient binary catalysts for the preparation of the corresponding products. The results demonstrated the better utility of PNU-21 (11–96%) in comparison to PNU-22 (8–85%).^100^

In 2023, a new adenine-functionalized dendritic fibrous nanosilica (DAD) was synthesized by functionalizing the surface of dendritic fibrous nanosilica (DFNS) with adenine using a bifunctional isocyanate crosslinker ((EtO)_3_Si(CH_2_)_3_NCO). This adenine-functionalized dendritic fibrous nanosilica was used as a bifunctional catalyst for CO_2_ fixation in order to react with epoxide (23) to achieve cyclic carbonates (25). The reaction proceeded in the presence of TBAB (tetrabutylammonium bromide) co-catalyst under solvent-free conditions.^101^ In 2005, the cycloaddition of CO_2_ to epoxides (epichlorohydrin, propene oxide, and styrene oxide) to afford cyclic carbonates (25) occurred in the presence of adenine-modified Ti-SBA-15 catalysts. In addition, carbamates were synthesized through the reaction of alkyl/aryl amines, CO_2_, and *n*-butyl bromide. In the synthesis of cyclic carbonates, the reaction proceeded without any additional co-catalysts like *N*,*N*-dimethylaminopyridine (DMAP) or quaternary ammonium salts. The process using the present catalyst system avoids hazardous substances like phosgene or isocyanate and progresses at low temperatures and pressures.^102^

**Scheme 9:**
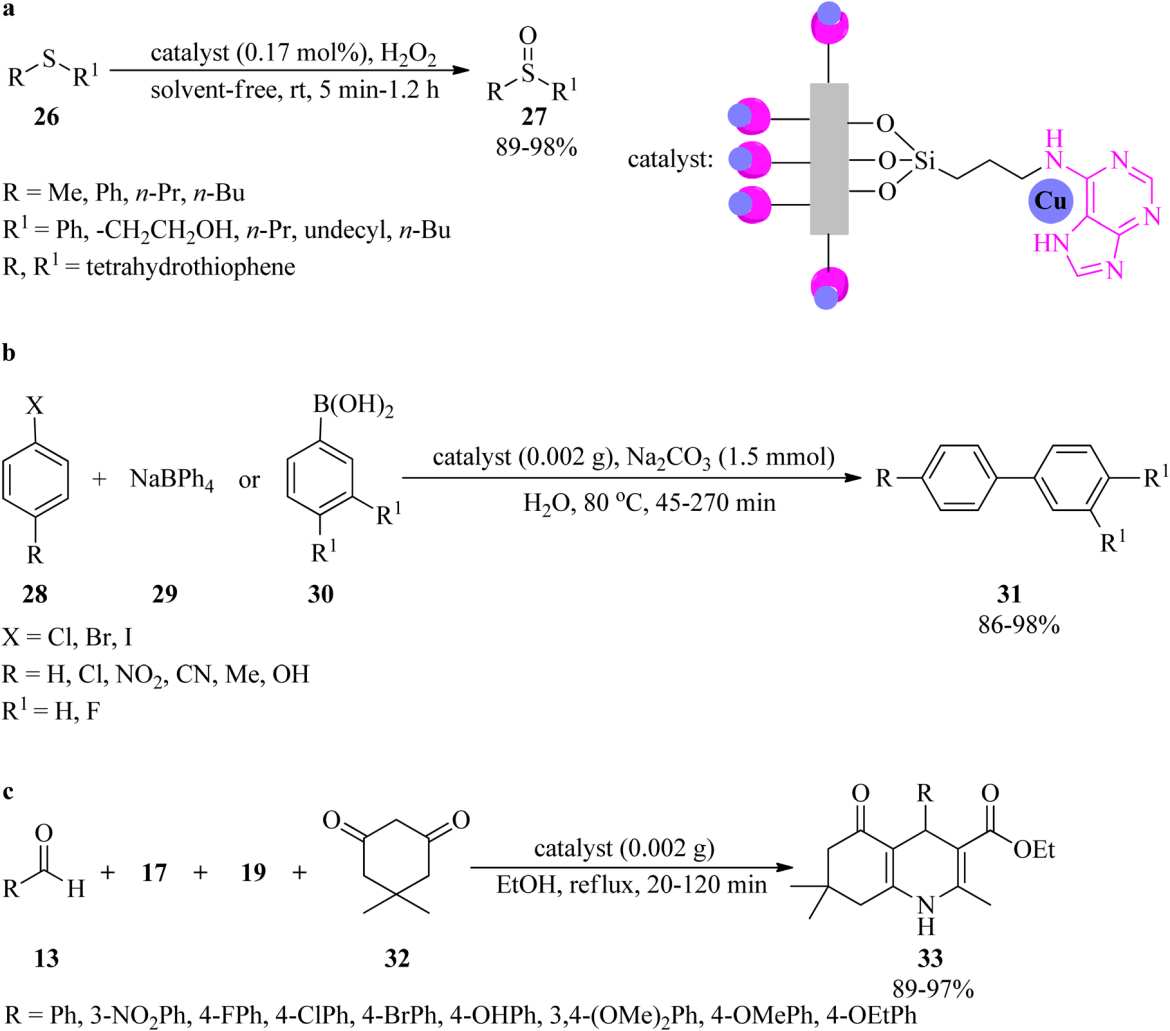
Various organic reactions in the presence of Cu-adenine@boehmite; (a) selective oxidation of sulfides to sulfoxides, (b) Suzuki coupling, and (c) synthesis of polyhydroquinolines.

Rigid copper on adenine-coated boehmite nanoparticles (Cu-adenine@boehmite) was prepared by Ghorbani-Choghamarani *et al.* in 2019, which catalyzed various organic transformations, such as the selective oxidation of sulfides (26) to sulfoxides (27) by hydrogen peroxide as an oxidant under solvent-free conditions at room temperature (Scheme 9a). On the basis of the resulting data, diverse aromatic, aliphatic, and heterocyclic sulfides indicated no significant difference in the reaction yields, turnover numbers (TON), and turnover frequency (TOF) values. Generally, the aromatic sulfides prolong the reaction times. It is noteworthy that this transformation was not accompanied by forming sulfone as the by-product.^103^ The adenine-containing catalyst also promoted the aqua-mediated formation of biphenyls (31) through the Suzuki C–C coupling reaction of aryl halides (28) with sodium tetraphenylborate (29) or phenylboronic acid derivatives (30) (in 1 : 0.5 and 1 : 1 molar ratios, respectively) utilizing sodium carbonate (Na_2_CO_3_) as a base at 80 °C (Scheme 9b). Notably, the coupling of with aryl iodides was performed in shorter reaction times than other aryl halides (chloride and bromide). The selectivity of the catalyst was examined *via* the utilization of 1-bromo-4-chlorobenzene in the coupling reaction with phenylboronic acid and sodium tetraphenyl borate. The results affirmed that the chloro functional group remained intact and the Suzuki reaction occurred on the bromo functional group as the only product. The formation of polyhydroquinolines (33) in aqueous media *via* the four-component condensation reaction of benzaldehyde (13), ethyl acetoacetate (17), ammonium acetate (19), and dimedone (32) in a 1 : 1 : 1.3 : 1 molar ratio, utilizing Cu-adenine@boehmite, were also performed successfully (Scheme 9c).^103^

**Scheme 10:**
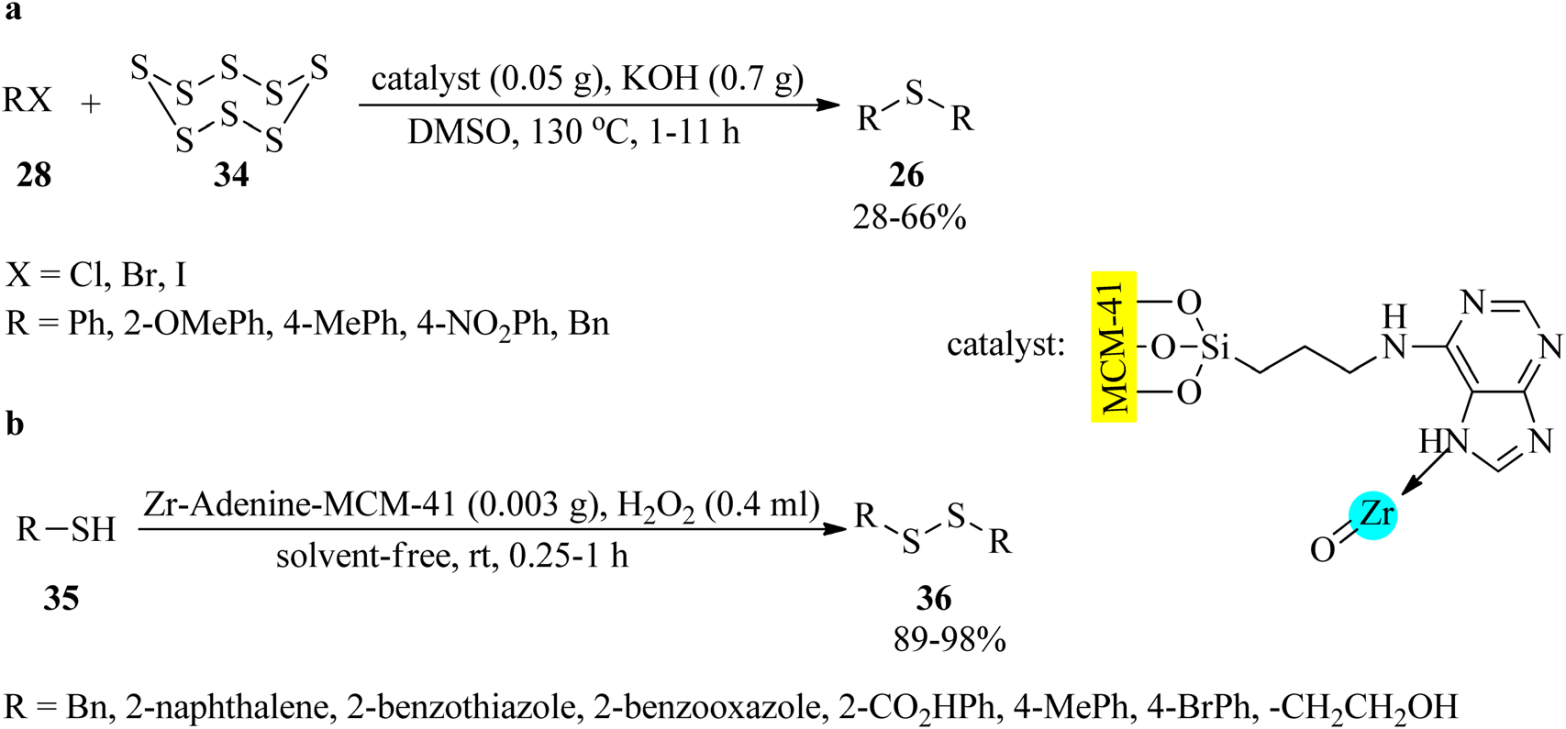
(a) Synthetic route to symmetrical sulfides, and (b) oxidative coupling reaction of thiols to disulfides in the presence of Zr-adenine-MCM-41.

In 2018, Tamoradi *et al.* designed and characterized a zirconium complex of adenine coated on mesoporous silica MCM-41 (Zr-adenine-MCM-41) as a non-toxic and thermally stable nanocatalyst to accelerate the synthesis of symmetrical sulfides (26) *via* the dimethyl sulfoxide-mediated reaction of aryl halides (28) with sulfur (34) in equimolar amounts (Scheme 10a).^104^ As illustrated in Scheme 10b, the oxidative coupling reaction of thiols (35) was also accomplished to afford disulfides (36) using catalytic amounts of Zr-adenine-MCM-41 by H_2_O_2_ as the oxidative reagent under solvent-free conditions at room temperature in 89–98% yields and very short reaction times.^104^ Solvent-less oxidation of diverse kinds of sulfides (26) to their corresponding sulfoxides (27) was also performed in the presence of MCM-41-adenine-Zr (0.004 g) at ambient temperatures using H_2_O_2_ oxidant (0.4 mL) in short reaction times (5–65 min) and excellent yields (89–97%).^104^ The recovery and the reusability test of Zr-adenine-MCM-41 demonstrated good results for the 3-run synthesis of sulfides and 6-cycles for the oxidation of sulfides and oxidative coupling of thiols. The observation described negligible leaching of zirconium from MCM-41-adenine-Zr.

Tamoradi *et al.*, in 2017, prepared a novel and green catalyst through the immobilization of Ni on magnetite nanoparticles coated with adenine (Fe_3_O_4_-adenine-Ni). The activity of the nanostructure was examined for the oxidation of sulfides (26) to sulfoxides (27) and oxidative coupling of thiols (35) to their corresponding disulfides (36) in the presence of H_2_O_2_ oxidant (0.5 mL). The reactions were performed at ambient temperature under solvent-free conditions and ethanol media to achieve the desired products in short reaction times (12–70 min, 25–90 min) and excellent efficacy (94–98%, 87–97%).^105^ The synthesis of polyhydroquinolines (33) *via* the refluxing ethanol-mediated four-component reaction of benzaldehydes (13), ethyl acetoacetate (17), ammonium acetate (19), and dimedone (32) in a 1 : 1 : 1.2 : 1 molar ratio, utilizing Fe_3_O_4_-adenine-Ni (0.05 g) was also performed successfully (145–255 min, 91–97%).^105^

**Scheme 11:**
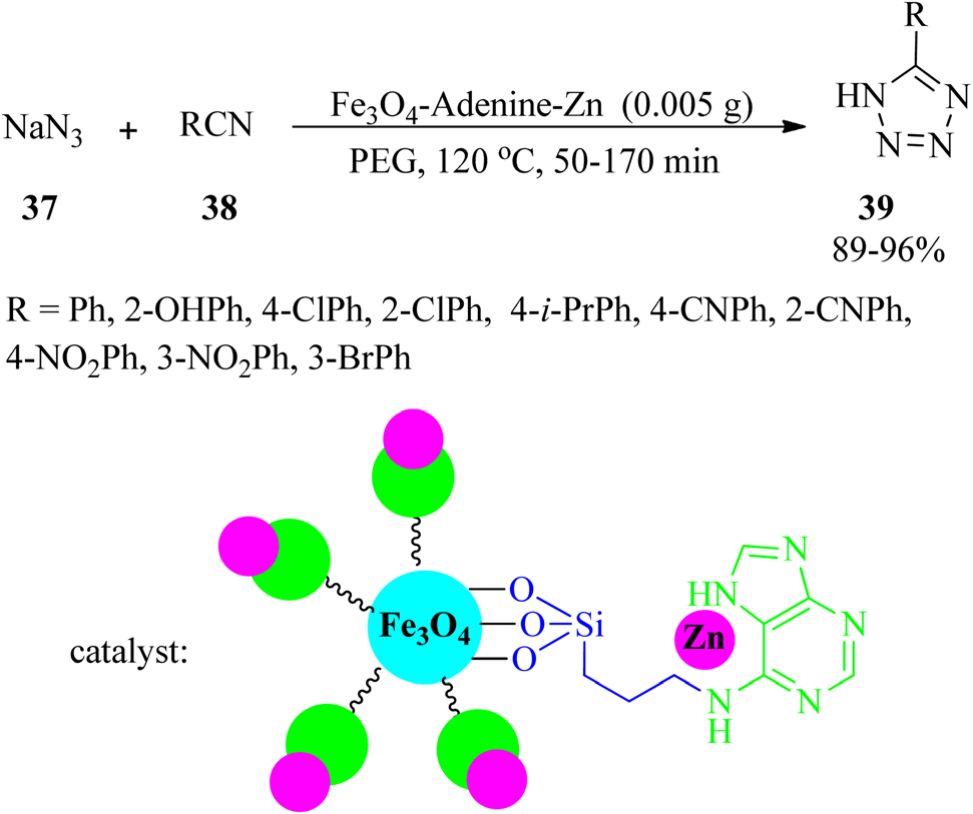
Synthesis of 5-substituted tetrazoles using sodium azide and benzonitriles.

Zinc(ii)-adenine complex functionalized on magnetite nanoparticles (Fe_3_O_4_-adenine-Zn) was synthesized and characterized in 2017. Its catalytic efficacy was tested for the synthesis of 5-substituted tetrazoles (39) *via* the reaction of sodium azide (37) with benzonitriles (38) in a 1.2 : 1 molar ratio (Scheme 11).^106^

The catalyst was also utilized to promote the solvent-free oxidation of sulfides (26) to sulfoxides (27) using H_2_O_2_ oxidant in 15–130 min with excellent yields (89–98%). The oxidative coupling of thiols (35) to their corresponding disulfides (36) also occurred in the presence of Fe_3_O_4_-adenine-Zn in ethyl acetate media at room temperature within 40–130 min by 86–99%.^106^ A wide range of thiols (aliphatic, aromatic, and heterocyclic) were successfully subjected to the mentioned oxidative reactions. This catalyst could be recovered easily and reused at least six times without significant loss of its catalytic activity.

**Scheme 12:**
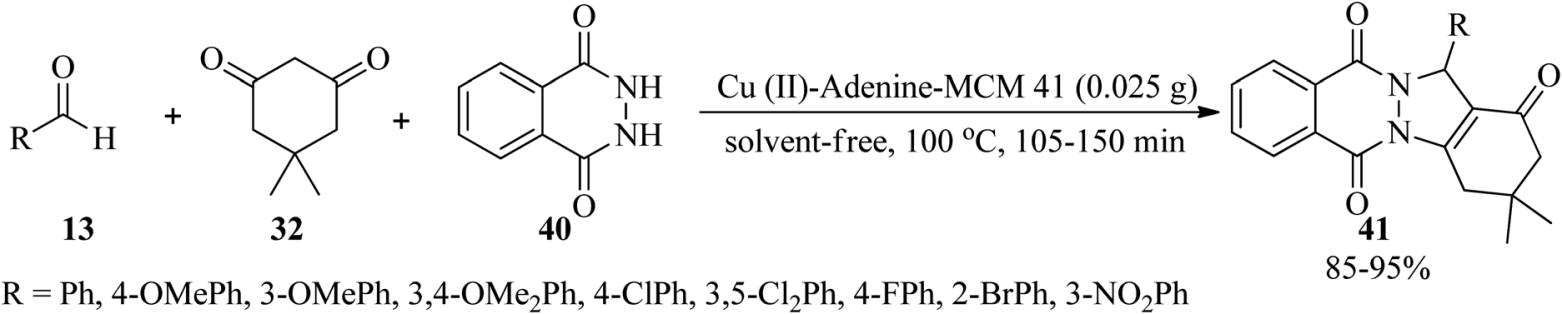
Synthesis of 1*H*-indazolo[1,2-*b*]phthalazine-triones.

5-Substituted 1*H*-tetrazoles (39) were also obtained through the reaction of sodium azide (37) and benzonitrile (38) in a 1.2 : 1 molar ratio in the presence of a Cu(ii) complex supported in MCM-41 channels modified with adenine (Cu(ii)-adenine-MCM-41 catalyst, 35 mg) in PEG-400 at 130 °C in 3–20 min and 70–92% yield.^107^ 1*H*-indazolo[1,2-*b*]phthalazine-triones (41) were also obtained through the solvent-free reaction of various benzaldehydes (13), dimedone (32), and phthalhydrazide (40) in a 1 : 1.2 : 1 molar ratio at 100 °C (Scheme 12).^107^ The catalyst is a Cu(ii)-Schiff-base complex supported on MCM-41, which was successfully prepared *via* the post-grafting method.

**Scheme 13:**
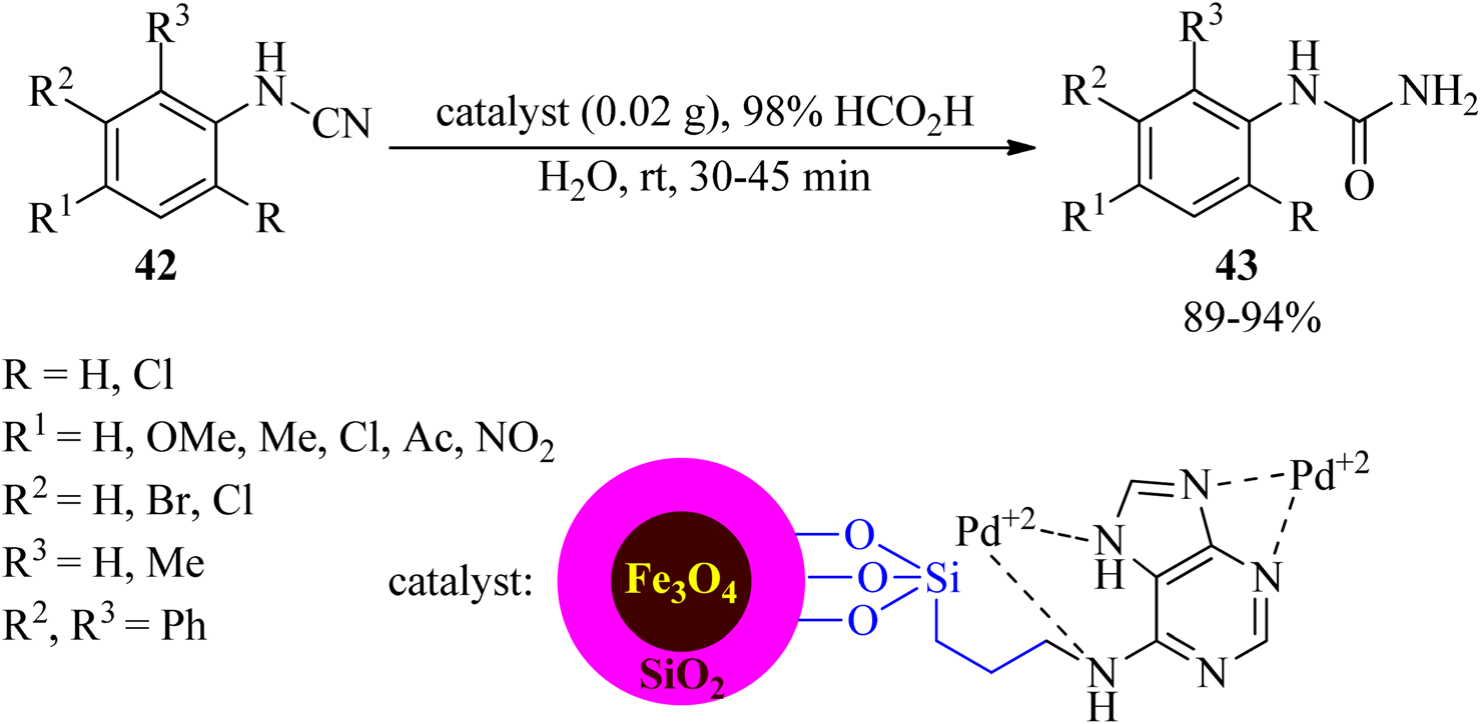
Generation of *N*-mono-substituted ureas.

In 2023, Khorram Abadi *et al.* examined the hydrolysis of cyanamides (42) by formic acid in water media in the presence of palladium(ii)-adenine complex coated on the surface of a silica-modified magnetic catalyst by employing 3-chloropropyltrimethoxysilane (CPTMS) as a linker and (Fe_3_O_4_@SiO_2_@CPTMS@A@Pd), as a new, efficient, and recyclable heterogeneous nanocatalyst at room temperature to obtain *N*-mono-substituted ureas (43) in high yields and short reaction times (Scheme 13). The catalytic system progressed well with all kinds of aryl cyanamides containing electron-donating and electron-withdrawing substituents.^108^ The catalyst was effective for five consecutive runs. The role of formic acid is probably to protonate the –CN group to increase its polarity. Also, it could protonate water to give H_3_O^+^ and HCOO^−^ to assist Pd in hydrolyzing CN to CONH_2_.

**Scheme 14:**
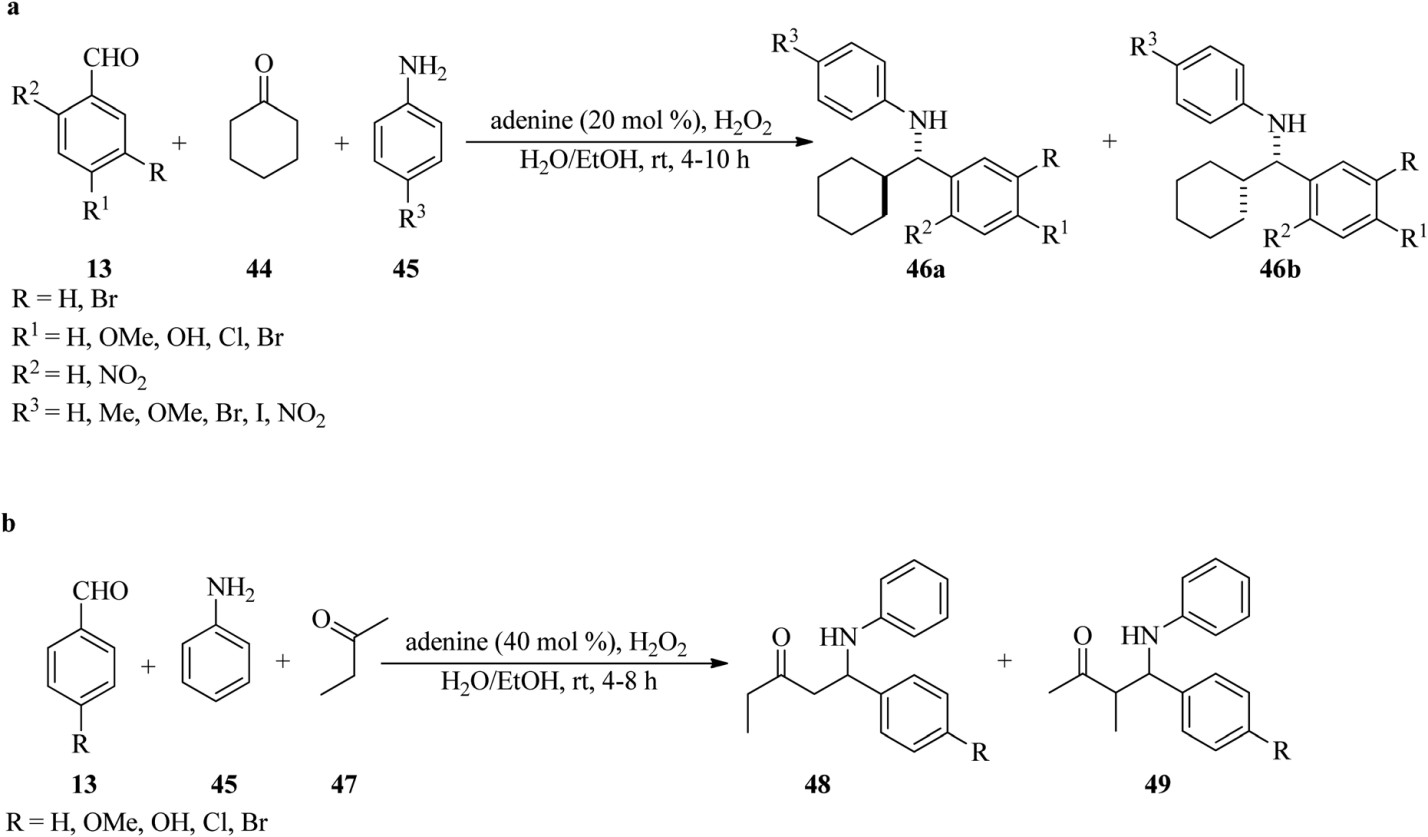
Adenine-mediated Mannich type reaction of (a) cyclohexanone, amines, and various benzaldehydes, and (b) 2-butanone, amines, and aldehydes.

In 2009, Goswami and Das developed an environmentally friendly approach for the diastereoselective synthesis of Mannich base products (46) *via* a three-component Mannich-type reaction of benzaldehydes (13), cyclohexanone (44), and anilines (45), utilizing adenine as an aminocatalyst (20 mol%) and H_2_O_2_ (30%, 4 μL) as additive in a mixture of water and ethanol (1/4) media at room temperature in 4–10 h in good to excellent yields (80–95%). It is significant that anilines, with electron-donating groups, resulted in the major generation of *anti*-products (46a) while the electron-withdrawing substituents (such as nitro and iodo) attained *syn*-form (46b) as the main products (Scheme 14a).^109^ It is necessary to mention that the condensation of cyclohexanone (47) and aniline, with both electron-donating and electron-withdrawing benzaldehydes, occurred successfully in high to excellent yields (75–93%). The stereochemical outcome appears to be unaffected, with the anti-isomer being the main product. Remarkably, the reaction with aliphatic aldehydes was unsuccessful. Replacing cyclohexanone with 2-butanone (47), as an unsymmetrical acyclic ketone, yielded two regioisomers 48 and 49, in which product 49 produced a mixture of diastereomers where the *anti*-diastereomers were the major products (Scheme 14b).^109^ In this transformation, the reactivity of 4-butanone was lower than that of cyclohexanone and 40 mol% of organocatalyst was needed for completion. The absence of column chromatography for majority of the compounds partially overcomes the major problem of epimerization of the Mannich products.^110^

In 2020, the rigid palladium-adenine complex on modified boehmite nanoparticles (Pd-adenine@boehmite) was introduced as a beneficial, recoverable, and reusable heterogeneous nanocatalyst for the aqua-mediated preparation of biphenyls (31) through the Suzuki coupling reaction of aryl halides (28) with sodium tetraphenylborate (29) or phenylboronic acid derivatives (30) utilizing sodium carbonate (Na_2_CO_3_) as the base at 80 °C within 0.5–4 h in high to excellent yields (85–96%).^111^ Notably, the resulting TOF values for aryl halides affirmed their reactivity as PhI > PhBr > PhCl. Furthermore, aryl halides bearing an electron-withdrawing group were more reactive. Sodium tetraphenylborate or phenylboronic acid derivatives have no significant influence on the yields. In addition, the [3 + 2] cycloaddition reaction of sodium azide (37) with benzonitriles (38) in PEG at 120 °C using Pd-adenine@boehmite resulted in 5-substituted tetrazoles (39) in 0.3–21 h and good to excellent yields (85–96%). Both electron-donating and electron-withdrawing benzonitriles resulted in products with good yields and appropriate TOF numbers.^111^

**Scheme 15:**
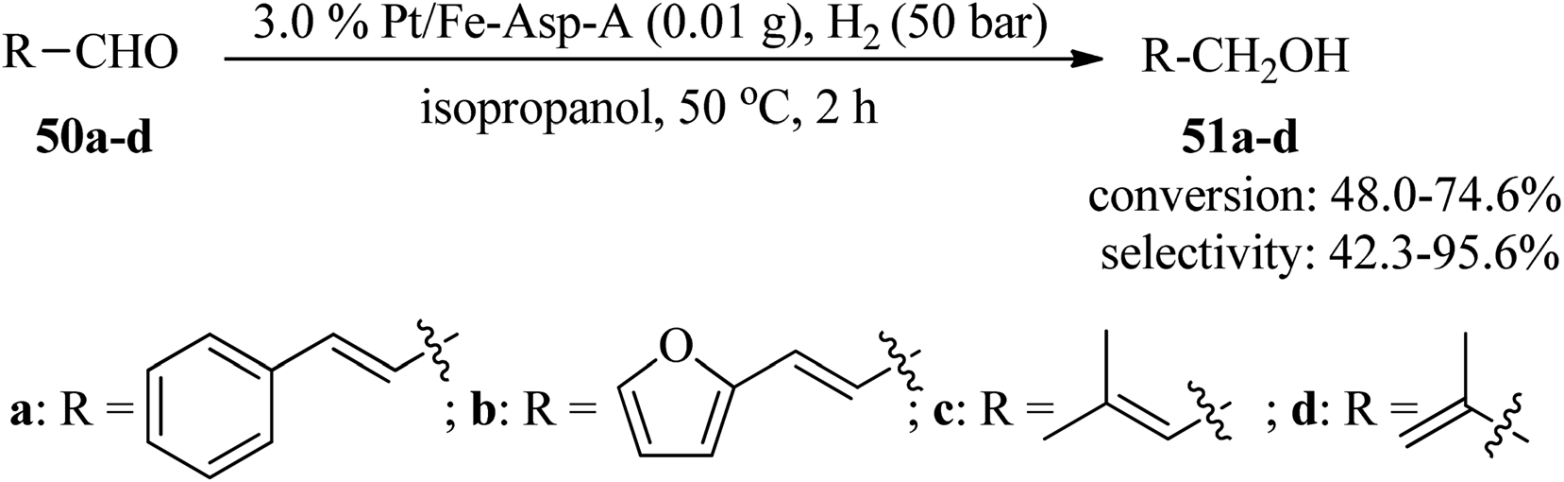
Selective hydrogenation of cinnamaldehyde to cinnamyl alcohol.

Cinnamyl alcohol is a significant chemical intermediate utilized in medicines, flavorings, and food additives. Cinnamyl alcohol is mainly produced through the selective hydrogenation of cinnamaldehyde.^112–114^ Hence, Tang *et al.*, in 2022, employed an amorphous Pt/Fe-Asp-A nanocatalyst for chemoselective hydrogenation of cinnamaldehydes. Adenine was utilized as a reinforcing agent to improve the chemical stability and selectivity of the Fe-l-aspartic (Fe-Asp) coordination material, which was modified through the loading of platinum nanoparticles on its surface. Finally, the resultant amorphous 3.0% Pt/Fe-Asp-A nanocatalyst catalyzed the reaction of cinnamaldehyde (50a) with hydrogen (H_2_) at 20 bar in the presence of isopropanol at 50 °C to furnish cinnamyl alcohol (51a) in a 2 h period with excellent selectivity (91.2%) and conversion (93.1%). The amorphous 3.0% Pt/Fe-Asp-A nanocatalyst also exhibited good performance to promote various α,β-unsaturated aldehydes (50a–d) under similar conditions with 48.0–74.6% conversion and 42.3–95.6% selectivity (Scheme 15). The recyclability and reusability of the synthesized nanocatalyst have been successfully investigated in up to ten runs without any considerable loss in catalytic activity.^115^

**Scheme 16:**
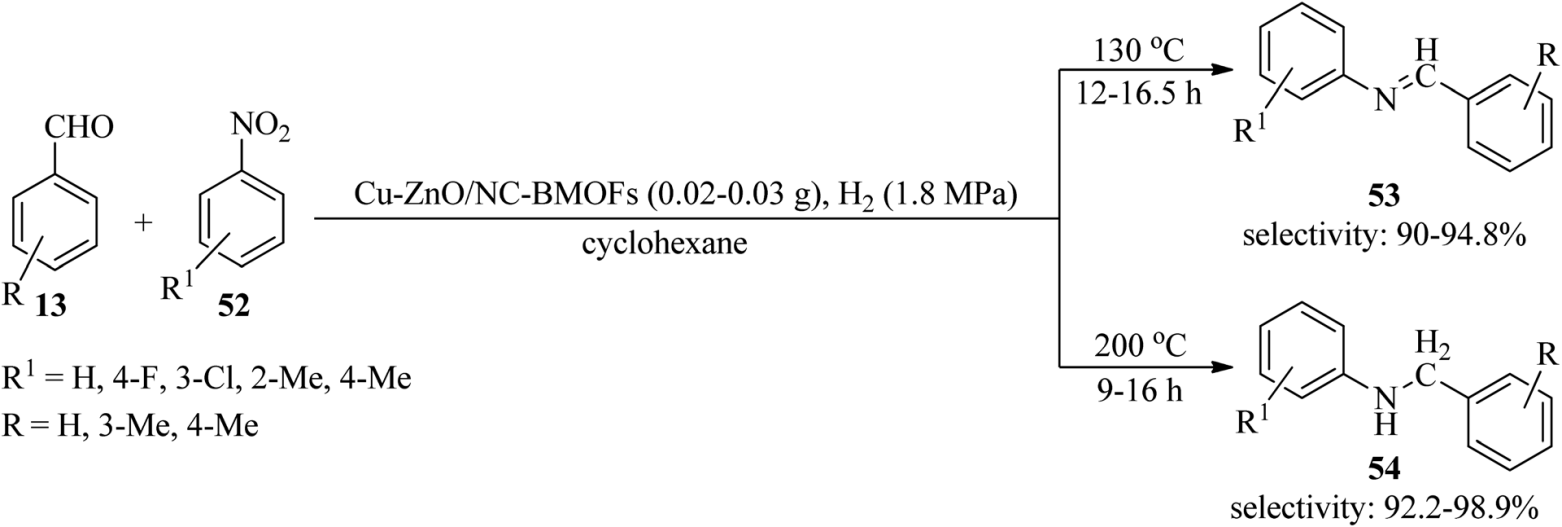
Synthesis of imines and secondary amines.

In 2021, a bimetallic CuZn-MOFs was generated *via* a facile solvothermal method utilizing adenine biomolecule as an organic linker. Moreover, a ZnO/nitrogen-doped carbon composite immobilized Cu catalyst (Cu-ZnO/NC-BMOFs) from CuZn-MOFs was obtained through a one-pot pyrolysis procedure. Then, its catalytic activity was examined by the hydrogenation/amination tandem reaction of benzaldehydes (13) and substituted nitroarenes (52) in cyclohexane *via* two pathways: (a) production of imines (53) at 130 °C in 12–16.5 h with high selectivity (90–94.8%), (b) generation of secondary amines (54) at 200 °C with high activity and high selectivity (92.2–98.9%) (Scheme 16). Comparatively, the Cu/ZnO/NC-IWI catalyst, prepared by incipient wetness impregnation, promoted the hydrogenation/amination reaction of nitrobenzene with benzaldehyde to give *N*-benzylaniline as compared to the CuZn-MOFs, and the conversion of nitrobenzene and selectivity toward *N*-benzylaniline was lower (49.6% and 10.9%). High selectivities, good stability, recyclability, and reusability of the catalyst are some of the advantages of this method.^116^

**Scheme 17:**
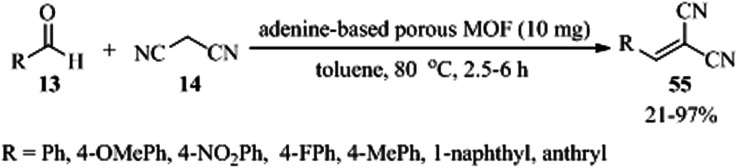
Knoevenagel condensation of malononitrile with different aldehydes.

A novel adenine-based porous MOF, named [H_2_N(CH_3_)_2_]·[Zn_4_(L)_1.5_(ad)_3_(H_2_O)_2_].4DMF (denoted as JUC-188, DMF = *N*,*N*-dimethylformamide), was prepared utilizing tetracarboxylic acid organic ligand, namely 5,5′-(1,3,6,8-tetraoxobenzo[Imn][3,8] phenanthroline-2,7-diyl)bis-1,3-benzenedicarboxylic acid (H_4_L), and adenine (ad) as organic linker. H_4_L and ad are both successfully connected to Zn(ii) ions. There are three different inorganic clusters in JUC-188, including ZnO_2_N_2_, Zn_2_O_2_N_6_, and ZnO_5_N clusters. The Knoevenagel condensation of different aldehydes (13) and malononitrile (14), in a 1 : 1.1 molar ratio, in the presence of JUC-188 as a solid catalyst has been examined successfully (Scheme 17).^117^ In the case of small aldehydes, the results are satisfactory. But for 1-naphthaldehyde and 9-anthracenecarboxaldehyde, the yields are very low, even with prolonged reaction times of 6 h with 43% and 21% yields, respectively. So, the catalyst is referred to as size-selective. Furthermore, it showed that adenine ligands can be applied to construct MOFs with the Lewis basic sites (–NH_2_) as heterogeneous catalysts.

**Scheme 18:**
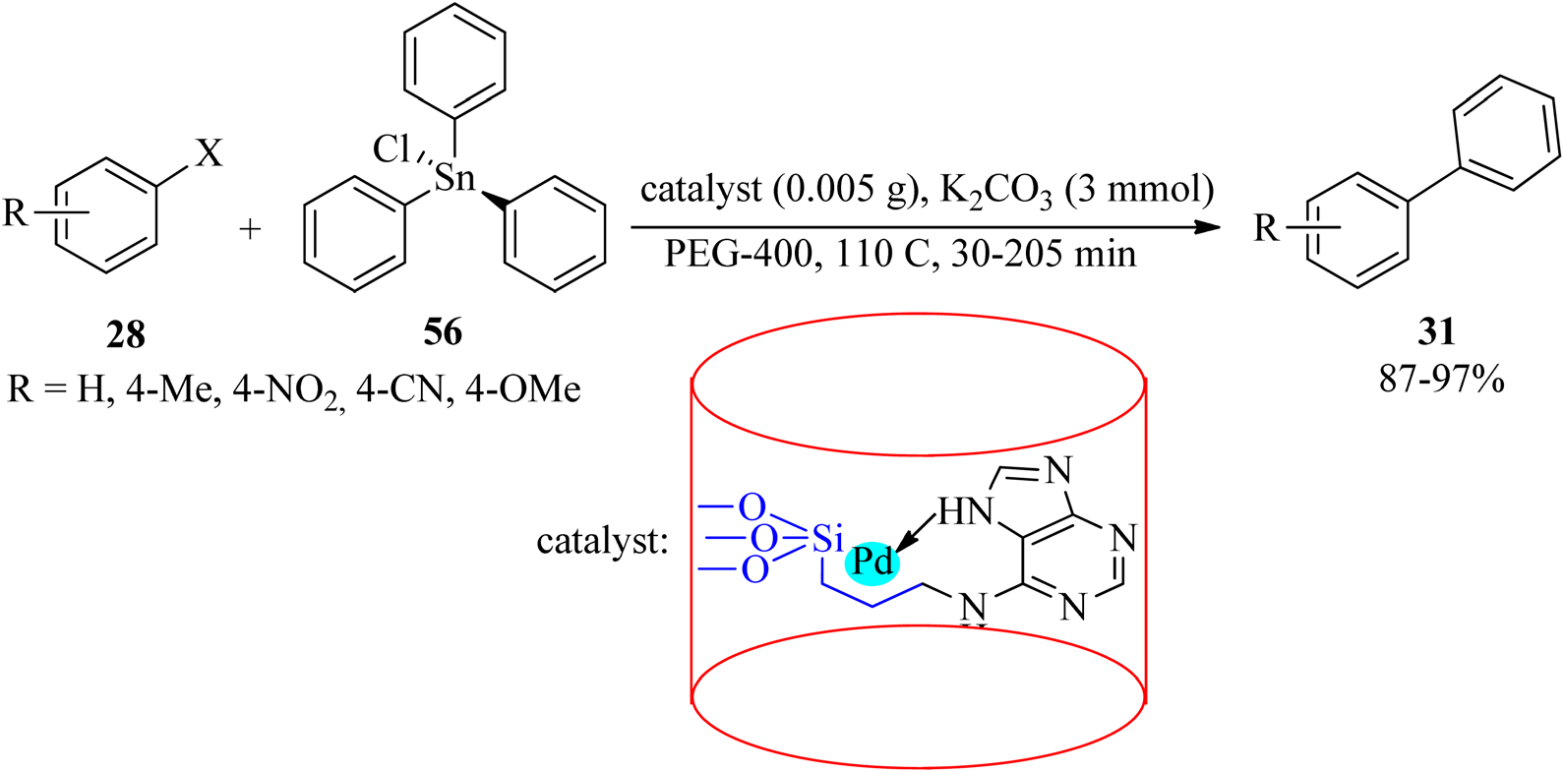
Stille cross-coupling reaction of aryl halides with triphenyltin chloride.

In 2019, palladium anchored on the surface of adenine-modified mesoporous silica was used to obtain SBA-15@adenine-Pd. Its efficacy was examined in the Stille cross-coupling reaction of different aryl halides (28) with triphenyltin chloride (56) with a 1 : 0.5 molar ratio in PEG-400 media at 110 °C (Scheme 18).^118^

The mesoporous silica-anchored SBA-15@adenine-Pd also promoted the Suzuki coupling of equimolar amounts of various aryl halides (28) with phenylboronic acid (30) in hot (110 °C) PEG-400 media in the presence of K_2_CO_3_ to obtain the corresponding biphenyl adducts (31) in 30–160 min and 83–98% yield.^118^ SBA-15@adenine-Pd was also effective for the synthesis of symmetrical sulfides (27) *via* the reaction of aryl halides (28) and sulfur (34) in DMSO at 130 °C in the presence of KOH (0.5 g) to afford the desired products within 80–610 min by 43–79%.^118^ The prepared catalyst separation could be easily achieved through filtration and drying after each run and can be reused for several consecutive cycles without a considerable decrease in its catalytic activity in all three types of the mentioned reactions.

**Scheme 19:**
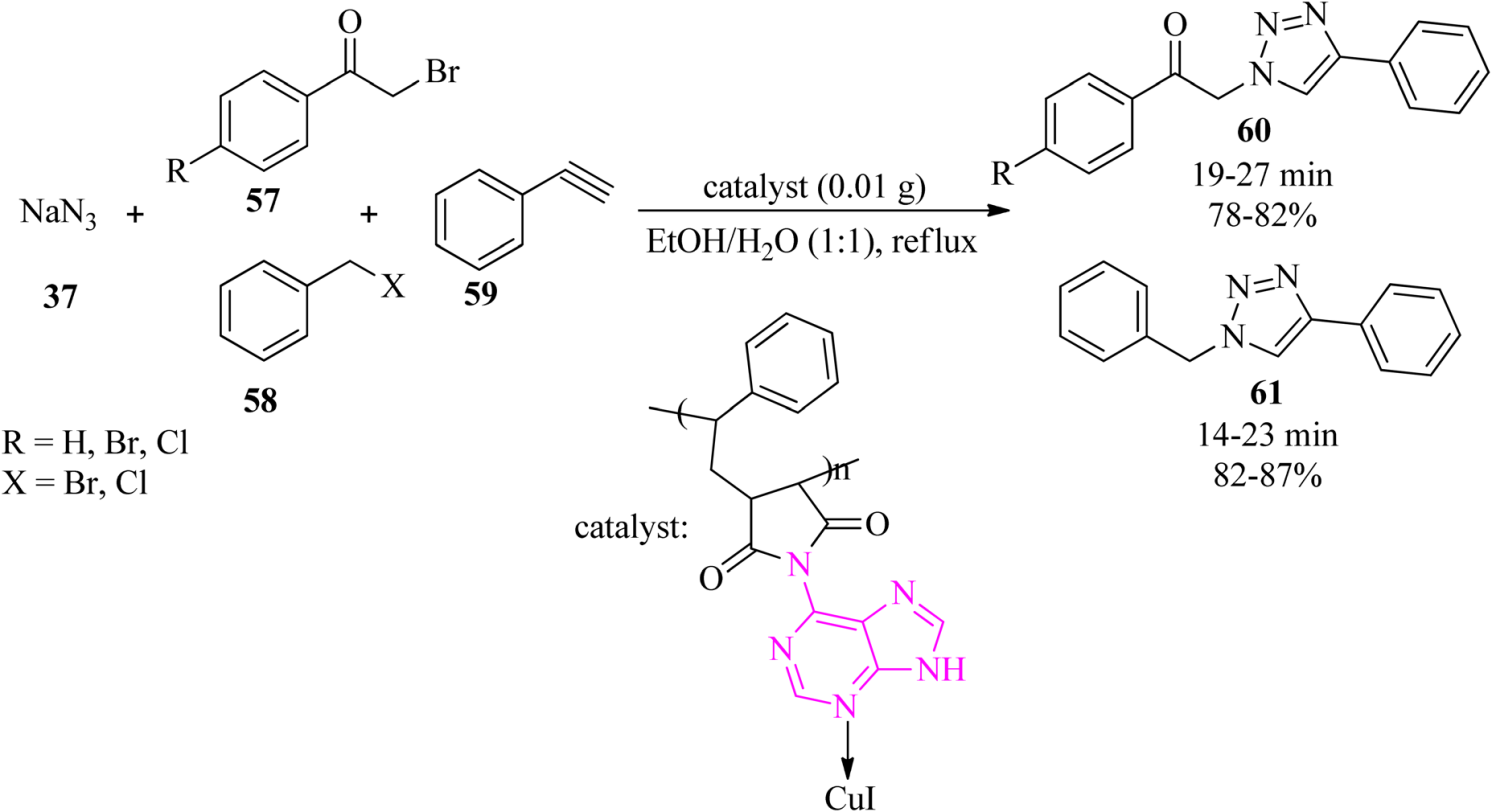
Synthesis of 1,4-disubstitued 1,2,3-triazoles *via* click reaction.

Novel adenine-based nano Cu(i) polymers were obtained *via* the immobilization of Cu(i) nanoparticles on modified poly(styrene-*co*-maleic anhydride) by adenine (Af-SMA-CuI). Its efficacy was successfully examined in the regioselective synthesis of 1,4-disubstituted 1,2,3-triazoles (60)/(61) *via* the click reaction of sodium azide (37), α-haloketones (57)/alkyl halide (58), and alkyne (59), in a 1.2 : 1:1 molar ratio, to give the corresponding products (60)/(61) in satisfactory yields (78–87%). The copper content in the catalyst was determined to be 23.83% (w/w), and each gram of the heterogeneous catalyst includes 1.25 mmol of copper (Scheme 19).^119^

**Scheme 20:**
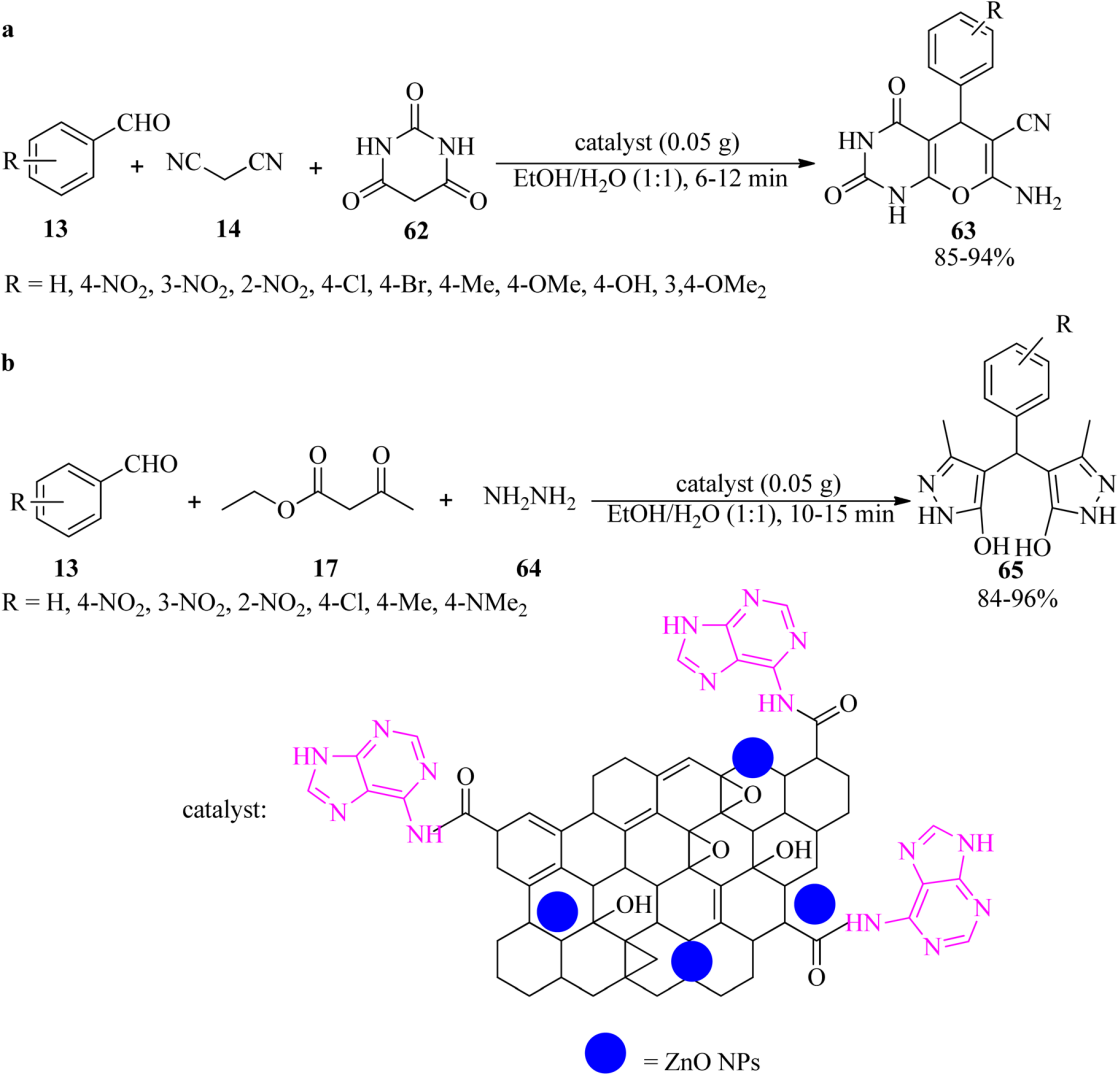
Synthesis of (a) pyrano[2,3-*d*]pyrimidines, and (b) bis(pyrazol-5-ole) derivatives.

In 2020, the adenine-grafted carbon-modified amorphous ZnO nanocatalyst (ZnO@AC) was derived from garment industry waste (waste cotton cloth). It promoted the synthesis of pyrano[2,3-*d*]pyrimidines (63) *via* the reaction of aldehydes (13), malononitrile (14), and barbituric acid (62) in EtOH/H_2_O at ambient temperature (Scheme 20a).^120^ The catalyst is also effective for the domino-type synthesis of bis(pyrazol-5-ole) derivatives (65) *via* the pseudo-five-component reaction of aromatic aldehydes (13), ethyl acetoacetate (17), and hydrazine hydrate (64), in a 1 : 2 : 2 molar ratio, at room temperature (Scheme 20b). The photocatalytic evaluation of ZnO@AC was performed on the methyl orange (MO) dye under UV light, with 87.3% degradation efficiency in 75 min. Moreover, the catalyst was recyclable and could be reused for up to eight runs, making it more sustainable.^120^

## 3. Catalytic activity of guanine in two- and multi-component reactions

**Scheme 21:**
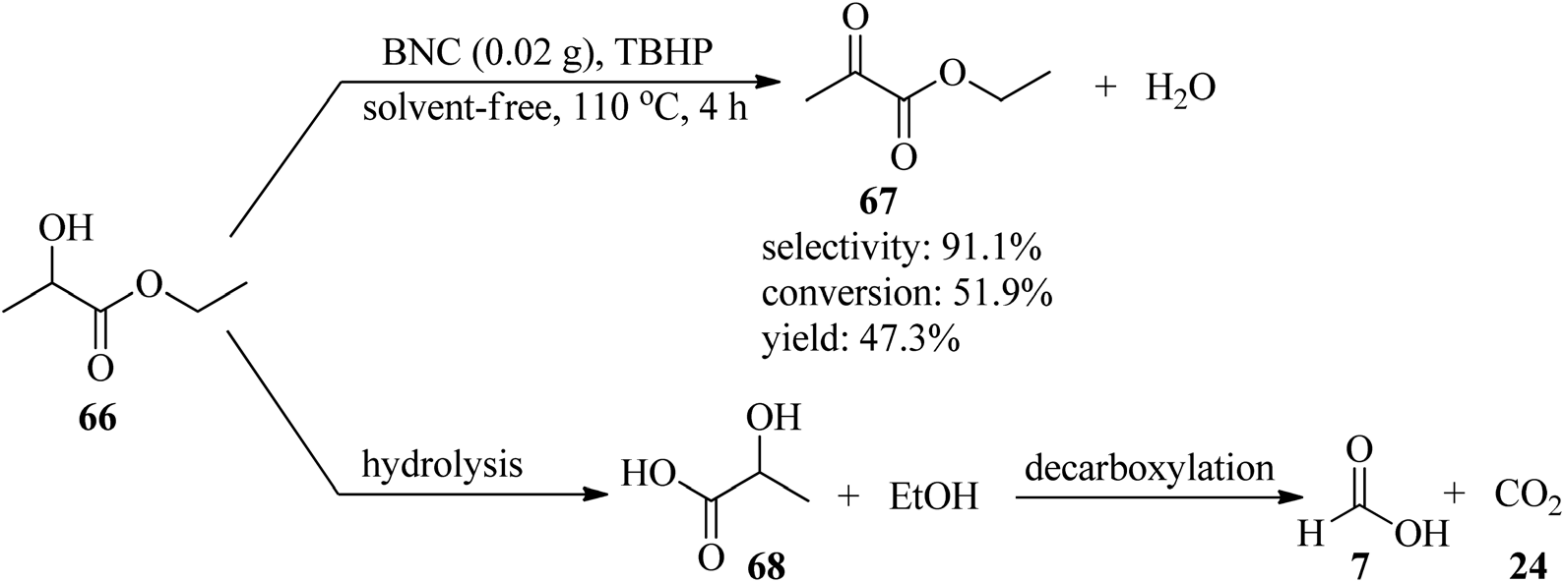
Preparation of ethyl pyruvate (EP) from ethyl lactate (EL).

In 2012, a cost-effective, metal-free, and template-free methodology was presented for the generation of boron (B) and nitrogen (N) co-doped carbon nanosheets (BNC), utilizing biomolecule guanine as the carbon (C) and N sources and boric acid as the B precursor. In the obtained BNC, guanine forms the G-quartet unit cells and expands to a larger planar network structure through multiple hydrogen bonds as both C and N sources. Subsequently, the resultant BNC catalyzed a liquid phase selective oxidation of ethyl lactate (EL, 66) to ethyl pyruvate (EP, 67) by *tert*-butyl hydroperoxide (TBHP) as an oxidant under solvent-free conditions at 110 °C for 4 h with excellent selectivity (91.1%) and moderate conversion (51.9%) and yield (47.3%). This transformation was accompanied by the production of ethanol and lactic acid (68) as by-products through hydrolysis, which could be attributed to the instability of the ester functional group at elevated temperatures. Then, decarboxylation of the resulting lactic acid yielded formic acid (7) and CO_2_ (24) (Scheme 21). On the other hand, the selective reduction of nitrobenzene (52) in the presence of hydrazine hydrate (64) under solvent-free conditions at 100 °C within 4 h afforded aniline (45) with excellent selectivity (95.5%), conversion (97.9%), and yield (93.5%). It is worth noting that in order to affirm the usefulness of the protocol, the oxidation and reduction reactions were carried out under catalyst-free conditions and in the presence of different catalysts such as graphene, nitrogen-doped carbon (NC), oxidized carbon nanotubes (oCNT), BNC-1 and BNC-2 (containing lower boric acid, and lower amounts of B, N and O), and according to the resulting data, the utilization of BNC was more efficient.^121^ The authors claimed that the guanine biomolecule leads to the formation of a graphitic structure during carbonization.

**Scheme 22:**
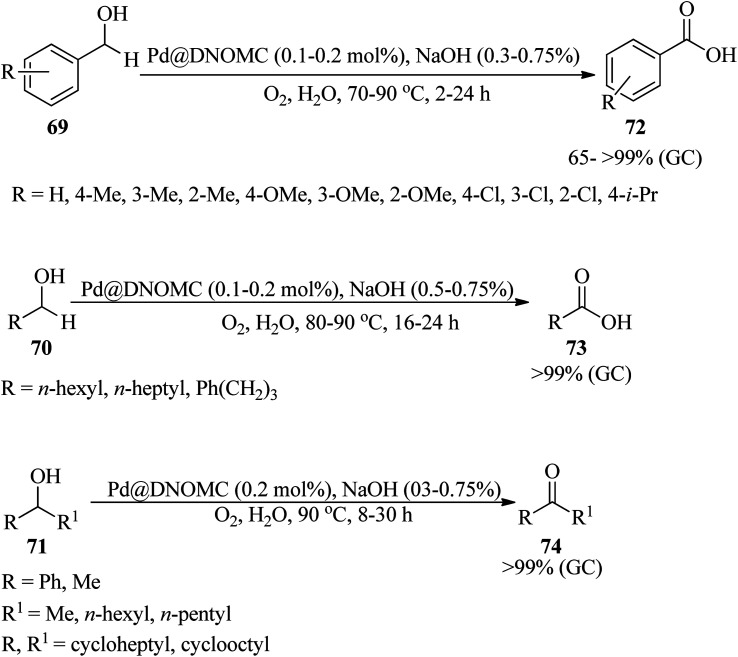
Aerobic alcohol oxidation using Pd@DNOMC.

In 2022, Alizadeh *et al.* reported for the first time the synthesis of DES-derived nitrogen-rich ordered mesoporous carbon (DNOMC) with a three-dimensional cubic framework utilizing highly ordered mesoporous silica KIT-6 (arising from pluronic P123, *n*-butanol, and (EtO)_4_Si) and a deep eutectic solvent (DES) bearing choline chloride and d-glucose as starting materials. The reaction progressed *via* the addition of guanine and urea as nitrogen sources to the resultant DES and subsequent carbonization by the KIT-6 template. The resulting DNOMC was employed as a powerful and efficacious support for the stabilization of palladium nanoparticles (Pd@DNOMC). Subsequently, the catalytic system proceeded *via* the aqua-mediated aerobic oxidation of diverse primary and secondary benzylic alcohols (69) as well as cyclic and acyclic aliphatic alcohols (70/71) to the corresponding carboxylic acids (72/73) (in 65 → 99% yields) and ketones (74) (>99% yields) within 2–30 h at 70 °C by NaOH in the presence of molecular oxygen (Scheme 22). High yields, low cost, easily available substrates, experimental accessibility, recoverability, and reusability of the catalyst for up to ten runs without any considerable loss of efficiency are some of the advantages of this strategy. In this research, the hot filtration test showed that the catalyst works through a boomerang-type catalyst route.^122^

**Scheme 23:**
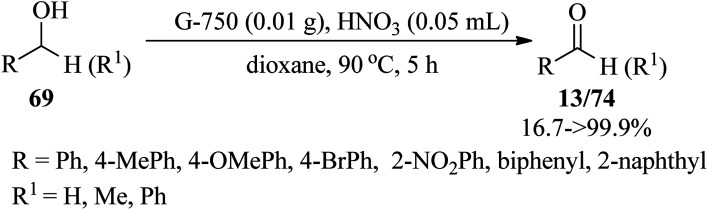
Selective oxidation of benzyl alcohols.

In 2023, Li *et al.* expressed a simple and metal-free procedure to synthesize *in situ* nitrogen-doped nanosheets through the pyrolysis of guanine at 750 °C, in which the guanine was chosen as the C and N precursor. The resulting *N*-doped nanocarbons (G-750) displayed a two-dimensional (2D) structure and high surface areas, which gave rise to excellent catalytic performance in the selective oxidation of benzyl alcohols (69) into benzaldehydes (13) or ketones (74) in 16.7%→99.9% yields by nitric acid as oxidant in 1,4-dioxane at 90 °C within 5 h (Scheme 23). It must be mentioned that graphitic nitrogen, pyridine nitrogen, and hydroxyl on the surface of nanocarbons play a key role in the reaction, in which the transformation of benzyl alcohol into benzaldehyde is mostly attributed to the concentration of hydroxyl groups, whereas the ratio of graphitic nitrogen and pyridine nitrogen has an important influence on the selectivity of benzaldehyde. Hence, the reactivity of benzyl alcohols bearing electron-donating groups was better than the alcohols with electron-withdrawing substituents, which could be ascribed to the inactivation of hydroxyl groups in catalytic reactions by the electron-withdrawing group.^123^

In 2019, Ng *et al.* prepared a novel palladium-guanine-reduced graphene oxide nanocomposite (Pd/rGO_G_) *via* an easy, scalable, one-pot microwave-assisted method, which introduced guanine to the reduced graphene oxide-supported palladium *via* non-covalent functionalization. The abundant amino, amide, and imino functional groups of guanine are considered to be the anchoring sites, which allow the uniform distribution of palladium nanoparticles (of various shapes, such as triangular, rectangular, circular, and diamond). The Pd/rGOG is an efficient catalyst for methanol oxidation. In addition, the guanine is revealed to be catalytically active toward the methanol oxidation reaction, serving as a second catalyst.^124^

**Scheme 24:**
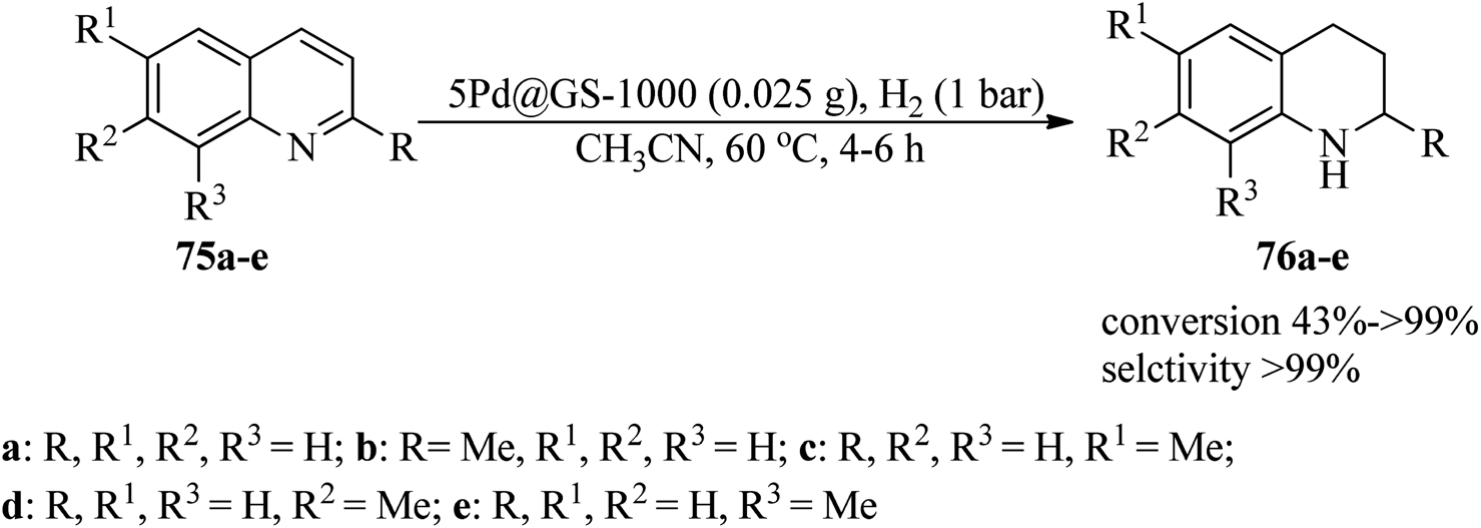
Hydrogenation of quinolones.

Hu *et al.* successfully designed a sustainable strategy for the synthesis of highly active and stable catalysts of N-doped and N/S-doped carbon nanosheet-coated palladium nanoparticles utilizing guanine or guanine sulfate as the nitrogen and carbon precursor, named Pd@G-1000 and Pd@GS-1000, respectively. The catalytic application was examined in the hydrogenation reaction of quinolones (75) under H_2_ (1 bar) in CH_3_CN solvent at 60 °C (Scheme 24). The 5Pd@GS1000 catalyst indicated considerably improved activity with >99% conversion for 1,2,3,4-tetrahydroquinoline (76a) in comparison with 5Pd@G-1000 with 63% conversion.^125^

**Scheme 25:**
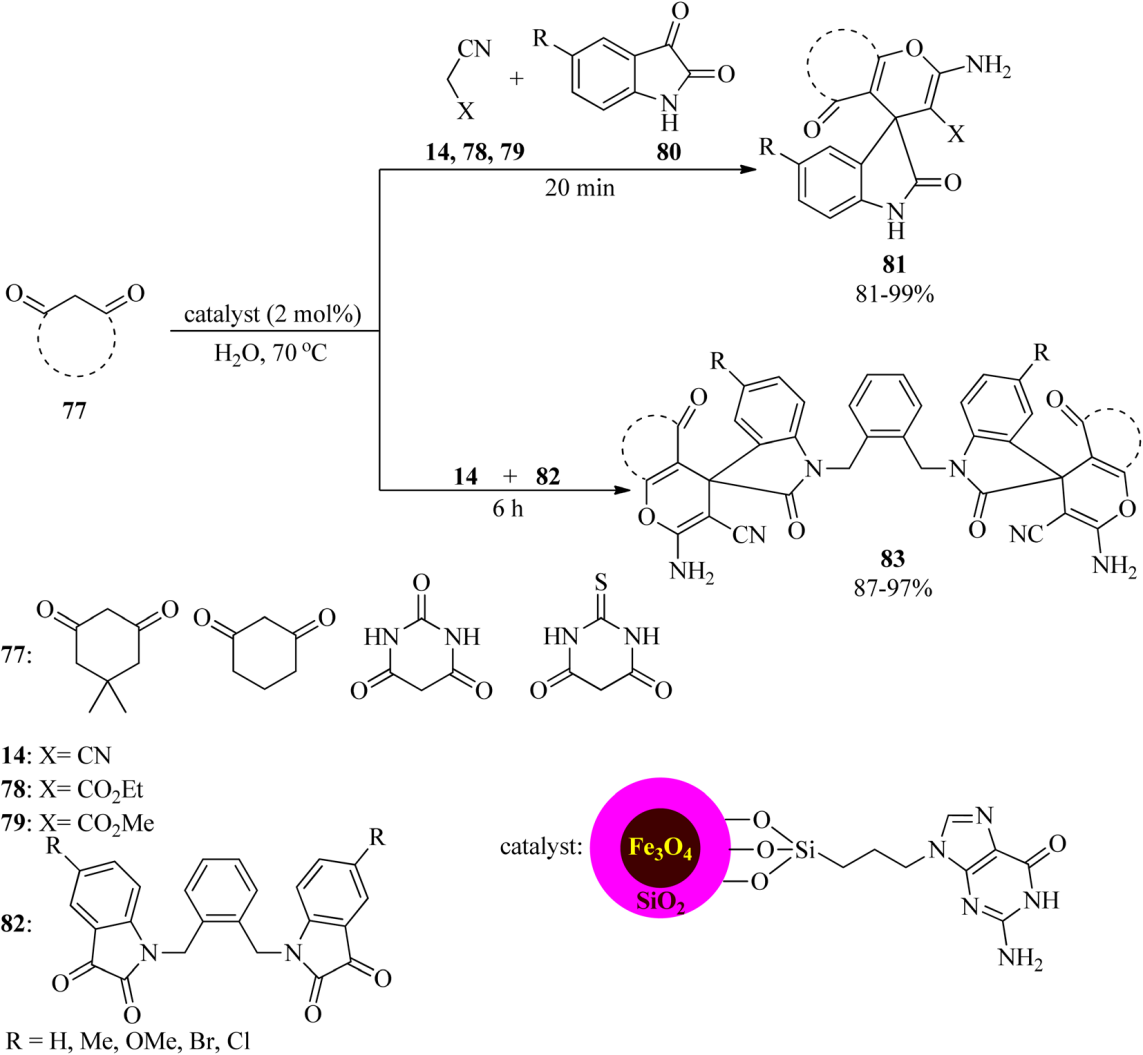
Preparation of 2′,5-dioxo-5,6,7,8-tetrahydrospiro[chromene-4,3′-indoline]-3-carbonitriles and substituted dihydro-2-oxopyrroles.

In 2021, Saberi *et al.* prepared and characterized a novel nanocomposite *via* guanine embedded on the surface of a silica-modified magnetic catalyst, using 3-chloropropyltrimethoxysilane (CPTMS) as a linker (Fe_3_O_4_@SiO_2_@CPTMS@guanine) and then its catalytic activity was investigated for the preparation of 2′,5-dioxo-5,6,7,8-tetrahydrospiro[chromene-4,3′-indoline]-3-carbonitriles (81) through the one-pot three-component reaction of equimolar amounts of 1,3-dicarbonyls (77), reactive methylene derivatives (14, 78, 79), and isatins (80) in aqueous media at 70 °C in 81–99% for 20 min. The magnetite nanoparticles (2 mol%) also catalyzed the aqua-mediated synthesis of substituted dihydro-2-oxopyrroles (83) by the one-pot multi-component reaction of 14, 1,3-dicarbonyls (77), and bis(isatin) derivatives (82), in a 2 : 2 : 1 molar ratio, at 70 °C (Scheme 25). Mild reaction conditions, easy separation of the catalyst from the reaction mixture by an external magnet, recyclability and reusability of Fe_3_O_4_@SiO_2_@CPTMS@guanine to promote the reaction for ten runs without any appreciable loss of efficiency, easy work-up, and excellent yields are some of the advantages of this procedure.^126^

**Scheme 26:**
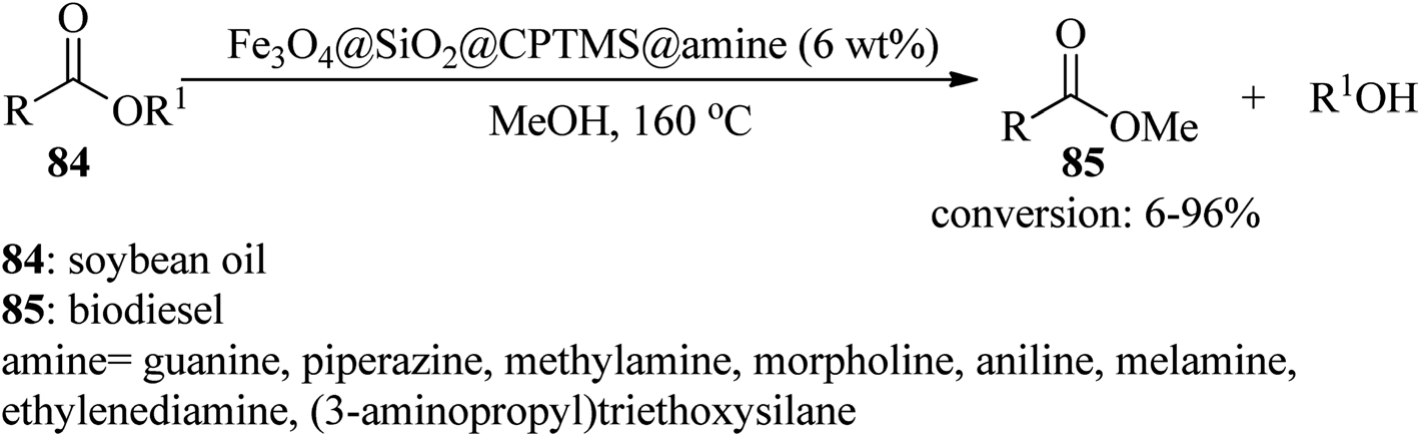
Biodiesel production from soybean oil.

In 2018, Farzaneh *et al.* produced several Fe_3_O_4_@SiO_2_@CPTMS@amine nanocomposites utilizing various amines, such as guanine, piperazine, methylamine, morpholine, aniline, ethylenediamine, 3-aminopropyltriethoxysilane, and melamine to afford biodiesel (85) with 6–96% conversions by the *trans*-esterification reaction of soybean oil (84) with methanol, in 1 : 36 molar ratio, at 160 °C for 3 h (Scheme 26). According to the resulting data, guanine and melamine gave the highest and lowest yields of the product, respectively, which is ascribed to the amine basicity of the catalyst.^127^

**Scheme 27:**
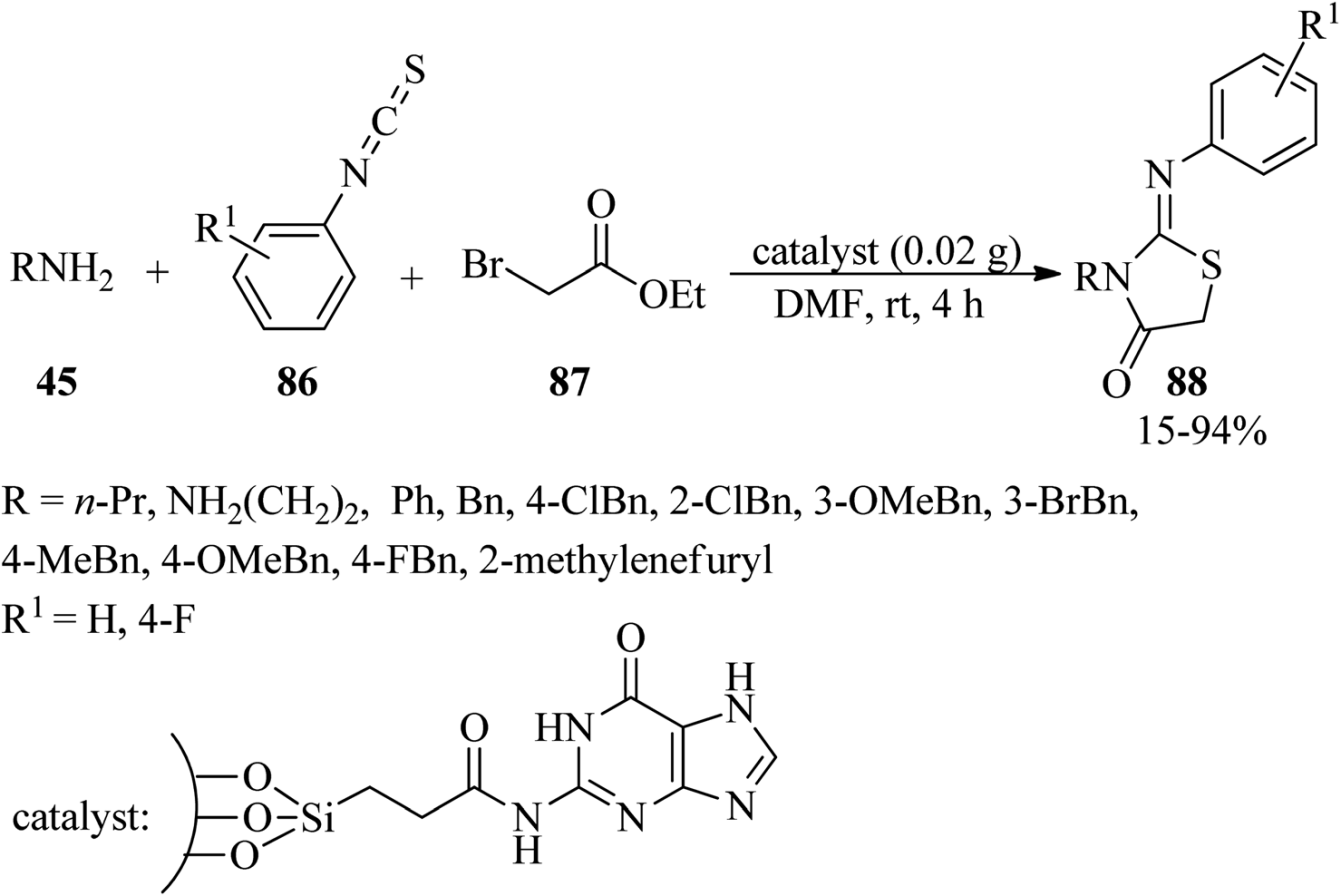
Synthesis of 2-iminothiazolidin-4-ones.

Due to the remarkable pharmaceutical activities of the heterocycles possessing thiazolidin-4-one motif, such as the antitubercular, antimicrobial, anticonvulsant, anticancer, antiprotozoal, and anti-inflammatory activities,^128,129^ Pathak and Gupta synthesized 2-iminothiazolidin-4-one derivatives (88) *via* the one-pot three-component annulation of amines (45), aryl isothiocyanates (86), and ethyl bromoacetate (87) utilizing guanine-coated SBA-16 (SBA-16@G) as an efficacious, practical, recyclable and reusable heterogeneous solid base catalyst in DMF at room temperature (Scheme 27). In this research, in order to probe the scope and limitations of the mentioned process, the reaction was performed using various aromatic and aliphatic amines such as aniline, benzylamines, ethylenediamine and propylamine. It was found that the reaction with benzylamines progressed faster. Remarkably, ethylenediamine and propylamine provided moderate yields (45% and 65%, respectively), while aniline gave poor yields (15%).^130^

**Scheme 28:**
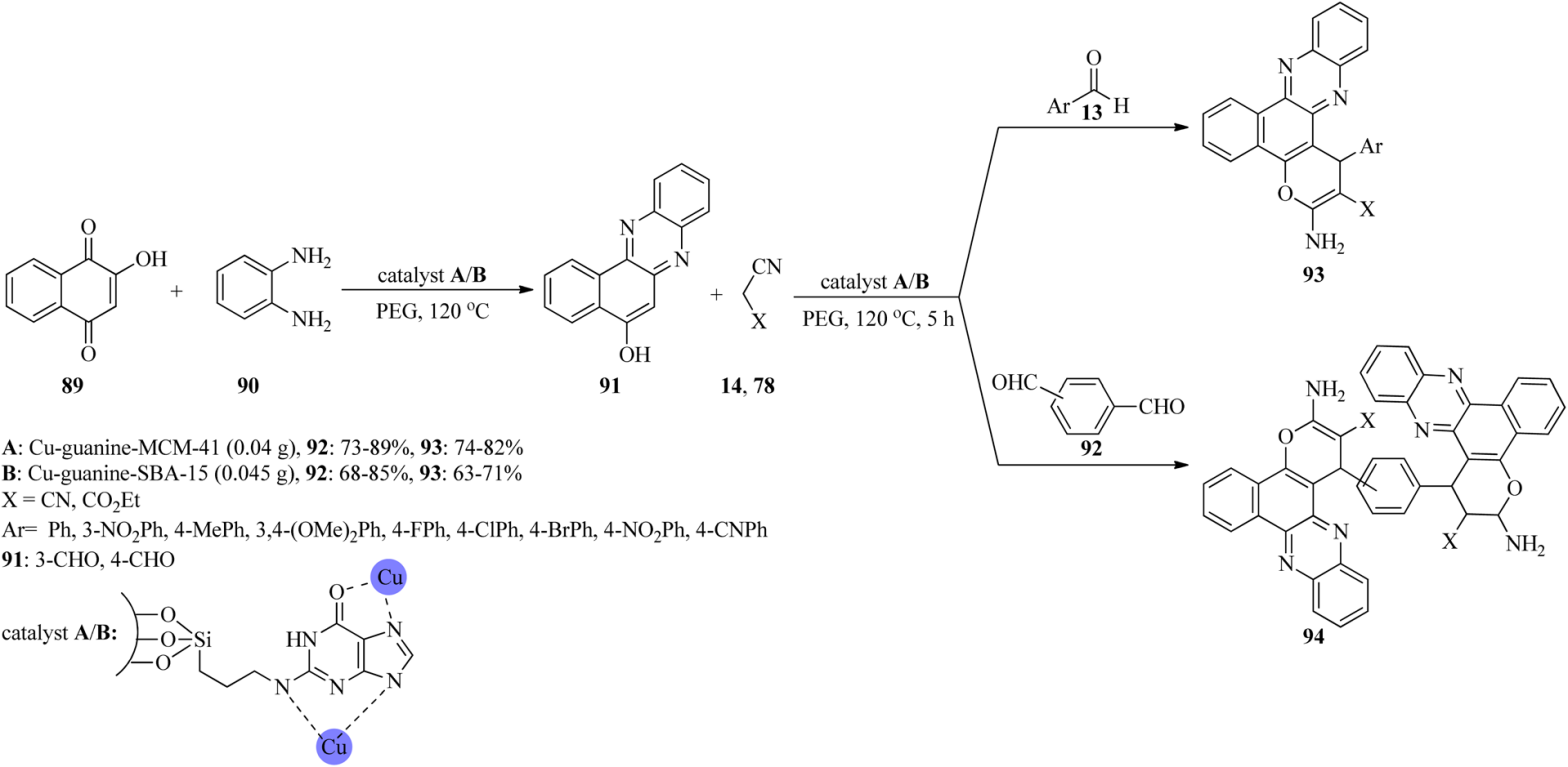
Generation of benzo[*c*]pyrano[3,2-*a*]phenazines and bis-benzo[*c*]pyrano[3,2-*a*]phenazines.

In 2020, novel copper(ii) ion complexes of guanine anchored on MCM-41 and SBA-15 (Cu-guanine-MCM-41 and Cu-guanine-SBA-15) channels were found to catalyze the generation of benzo[*c*]pyrano[3,2-*a*]phenazines (93) and bis-benzo[*c*]pyrano[3,2-*a*]phenazines (94) *via* the one-pot domino four-component reactions. These reactions began through the domino condensation reaction of 2-hydroxy-1,4-naphthoquinone (89) with benzene-1,2-diamine (90) in the presence of PEG at 120 °C to form phenazine (91), as the orange solid within 10 min, which was then treated with aldehydes (13, 92) and alkylmalonates (14, 78) to achieve the adducts 93/94 (Scheme 28). The reaction works well with aldehydes containing electron-donating groups. Furthermore, malononitrile resulted in higher yields in comparison to ethyl cyanoacetate. The results showed the yield was better utilizing Cu-guanine-MCM-41 (73–89%). Some of the products revealed antimicrobial activity against the growth of *Staphylococcus aureus*.^131^

**Scheme 29:**
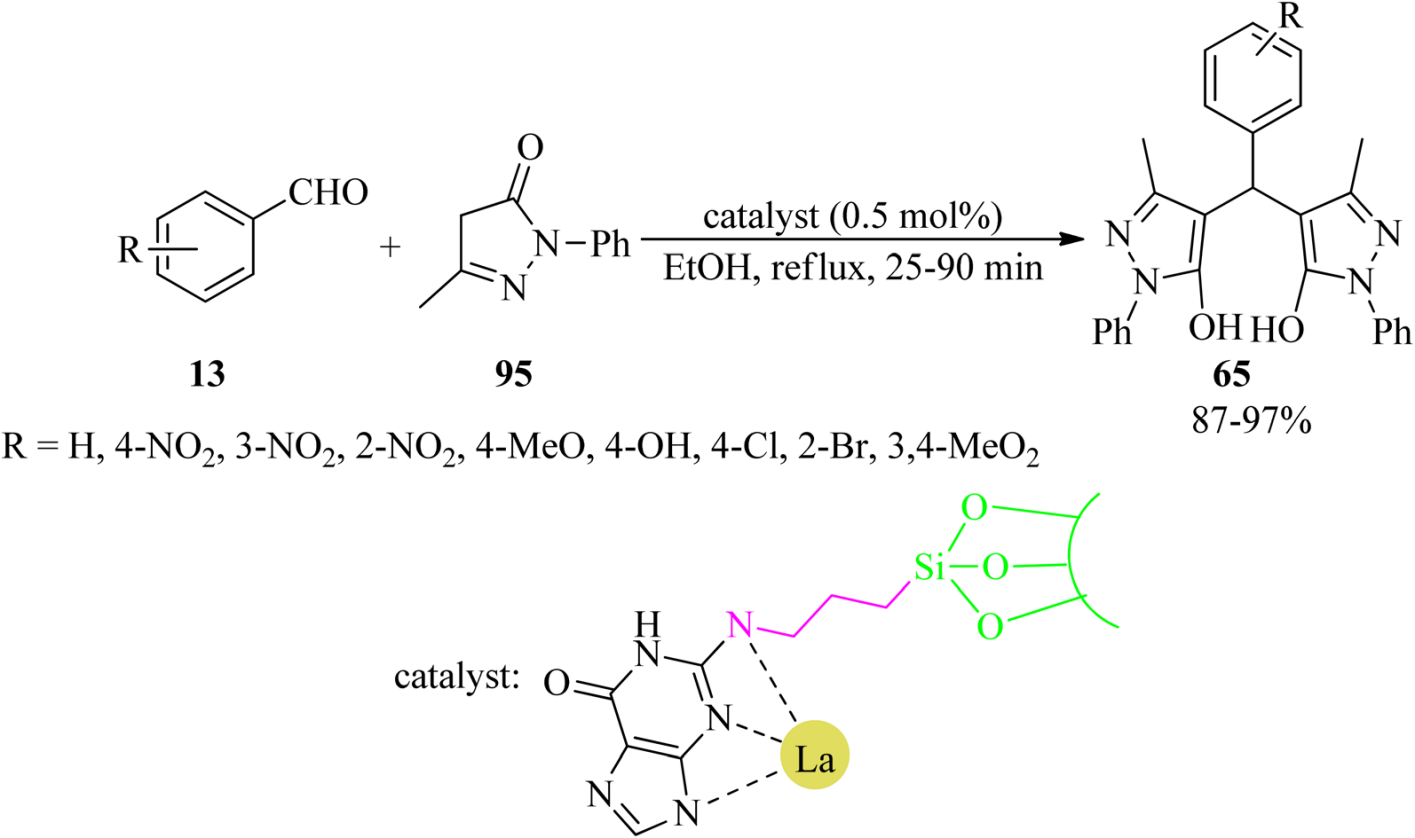
Synthesis of 4,4′-(arylmethylene)-bis-(3-methyl-1-phenyl-1*H*-pyrazol-5-ol) derivatives.

In 2020, Nikoorazm *et al.* obtained a novel lanthanum(iii) organometallic complex through a guanine-La complex embedded in SBA-15 (La-guanine@SBA-15). The heterogeneous mesoporous nanocatalyst was utilized for the one-pot, multi-component tandem Knoevenagel condensation-Michael addition–cyclization reactions in order to prepare a series of benzo[*a*]pyrano[2,3-*c*]phenazines (93) *via* the domino reaction of 89, 90, 14/78, and aldehydes (13), in a 1 : 1 : 1.5 : 1 molar ratio, in refluxing ethanol in 2.5–5 h with 87–99% yields. La-guanine@SBA-15 was also effective to obtain 4,4′-(arylmethylene)-bis-(3-methyl-1-phenyl-1*H*-pyrazol-5-ols) derivatives (65) *via* the reaction of aldehydes (13) and 3-methyl-1-phenyl-1*H*-pyrazol-5(4*H*)-one (95), in a 2 : 1 molar ratio (Scheme 29).^132^ In addition, this nanocatalyst was easily recovered, using simple filtration, and reused several times without significant loss of catalytic efficiency (confirmed by SEM and FT-IR techniques). Moreover, the leaching, heterogeneity and stability of La-guanine@SBA-15 were studied using the hot filtration test and ICP techniques.

**Scheme 30:**
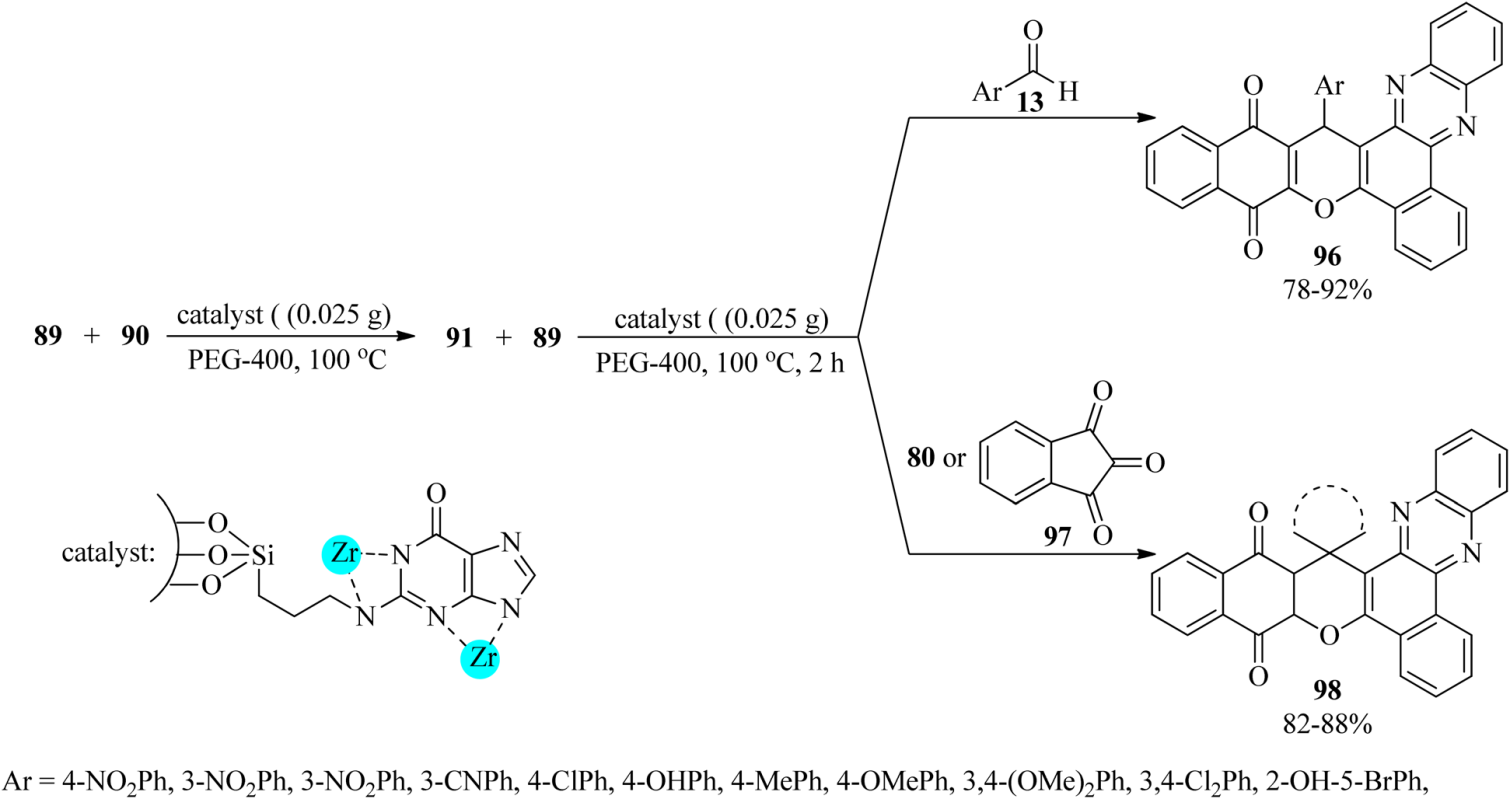
Preparation procedure of benzo[*α*]benzo^6,7^ chromeno[2,3-*c*] phenazines and spiro[benzo[*a*]benzo^6,7^ chromeno[2,3-*c*]phenazine] derivatives.

MCM-41-supported nanoscale guanine covered by Zr(IV) was prepared using the sol–gel method (Zr-guanine-MCM-41). This mesoporous nanostructure mediated the tandem chemoselective pseudo four-component reaction of 2-hydroxy-1,4-naphthoquinone (89), 90, and aldehydes (13), in a 2 : 1 : 1 molar ratio in PEG at 100 °C to produce benzo[*α*]benzo[6,7] chromeno[2,3-*c*]phenazines (96). The preparation of spiro[benzo[*a*]benzo[6,7] chromeno[2,3-*c*]phenazine] derivatives (98) was also performed through the pseudo-four-component reaction of 2-hydroxy-1,4-naphthoquinone (89), 90, isatins (80)/ninhydrin (97), effectively. These reactions progressed *via* the Schiff-base condensation reaction of 89 with 90 to generate the corresponding 91, which was treated subsequently with excess amounts of 89 and 13 or 80/97 (Scheme 30). Significantly, the reactivity of aldehydes, including electron-withdrawing groups, was better. The catalytic system also progressed well with aldehydes (13) and 3-methyl-1-phenyl-5-pyrazolone (95), in a 1 : 2 molar ratio, to furnish bis(pyrazolyl)methanes (65) in refluxing ethanol for 10–60 min in excellent yields (83–99%). Both electron-donating and electron-withdrawing aldehydes gave the products in good yields, but the reaction time of the aryl aldehydes containing electron-donating groups was longer.^133^

**Scheme 31:**
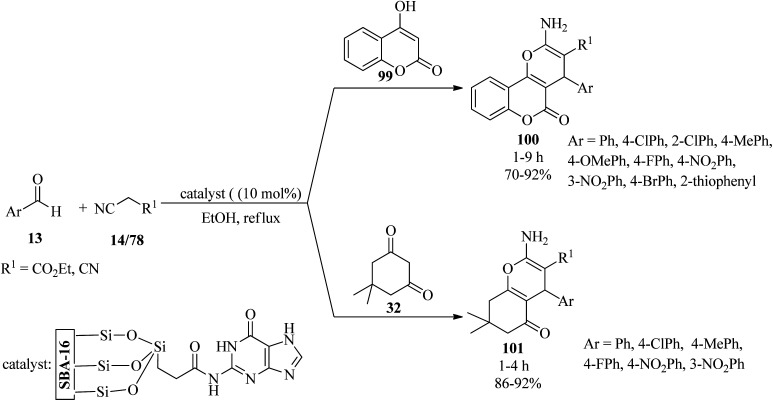
Synthesis of diverse pyran-annulated heterocyclic compounds.

In 2019, Gupta *et al.* synthesized guanine-functionalized mesoporous silica [SBA-16-G] with a surface area and basicity of 524 m^2^ g^−1^ and 3.230 mmol g^−1^, respectively. Its catalytic activity was explored in the synthesis of a series of pyran-annulated heterocyclic compounds 100/101 from a one-pot three-component reaction of aromatic aldehydes (13), malononitrile (14)/ethyl cyanoacetate (78), and C–H activated acidic compounds (dimedone (32)/4-hydroxycoumarin (99)) (Scheme 31).^134^ The special features of the protocol are a simple work-up procedure (no chromatography), recyclability up to four times, and the environmental acceptability of the catalyst due to metal-free catalysis.

**Scheme 32:**
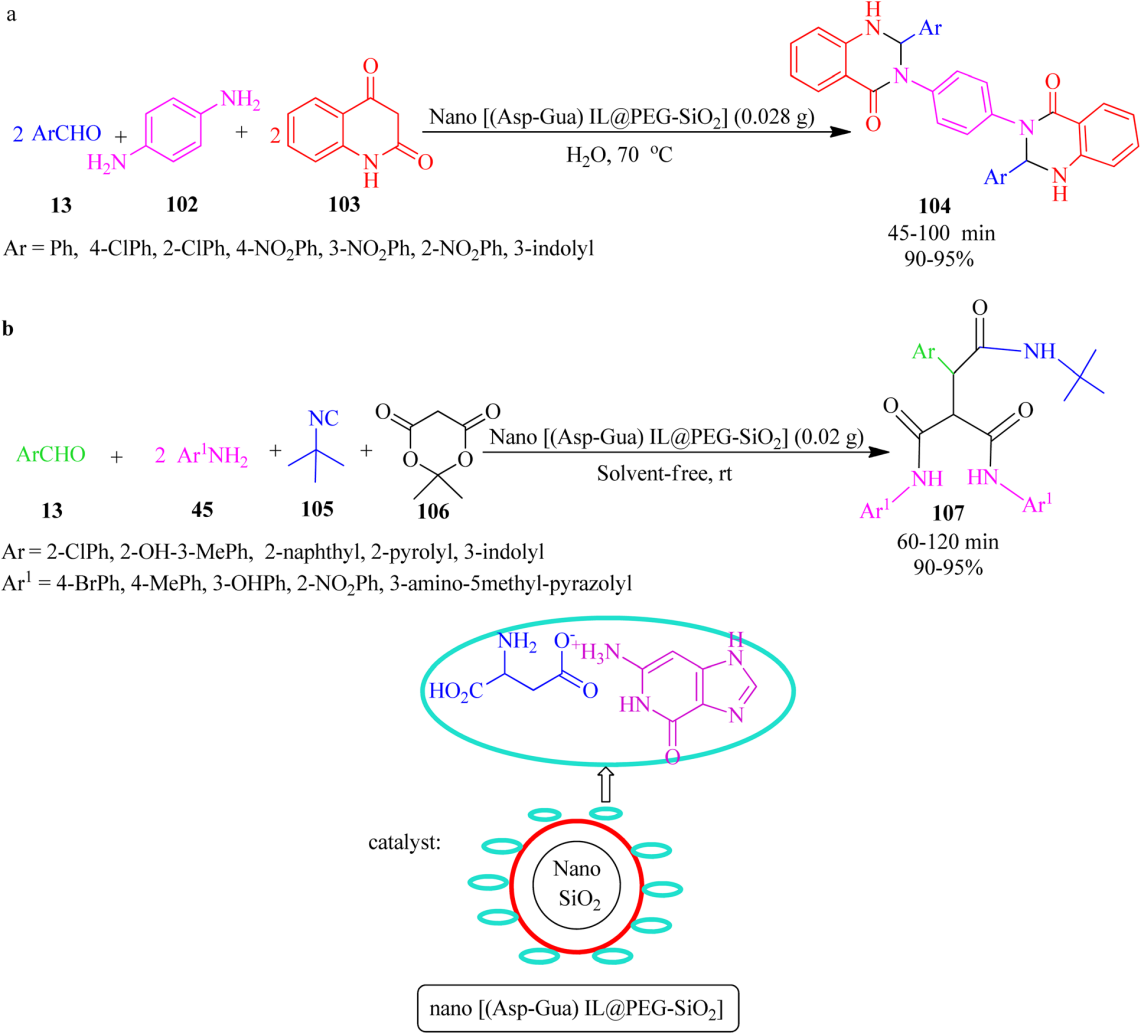
Synthesis of (a) bis(2,3-dihydroquinazolin-4(1*H*)-one) derivatives and (b) tricarboxamides.

In 2020, Nikoofar and Shahriyari prepared a novel bio-based core–shell organic–inorganic nanohybrid by embedding aspartic acid-guanine IL on the hydroxylated nanosilica surface (nano[(Asp-Gua)IL@PEG-SiO_2_]), as a versatile nanostructure hybrid for the synthesis of 2,3-dihydroquinazolin-4(1*H*)-one-derivatives (104) *via* the one-pot pseudo-five-component reaction of aldehydes (13), 1,4-benznendiamine (102) and isatoic anhydride (103) at 70 °C in aqueous media (Scheme 32a). In addition, the peptide-like tricarboxamides (107) were also achieved using the pseudo-five-component condensation of aromatic aldehydes (13), aromatic amines (45), *tert*-butyl isocyanide (105), and Meldrum's acid (106) under green solventless conditions (Scheme 32b).^135^

## 4. Catalytic activity of cytosine in two- and multi-component reactions

**Scheme 33:**
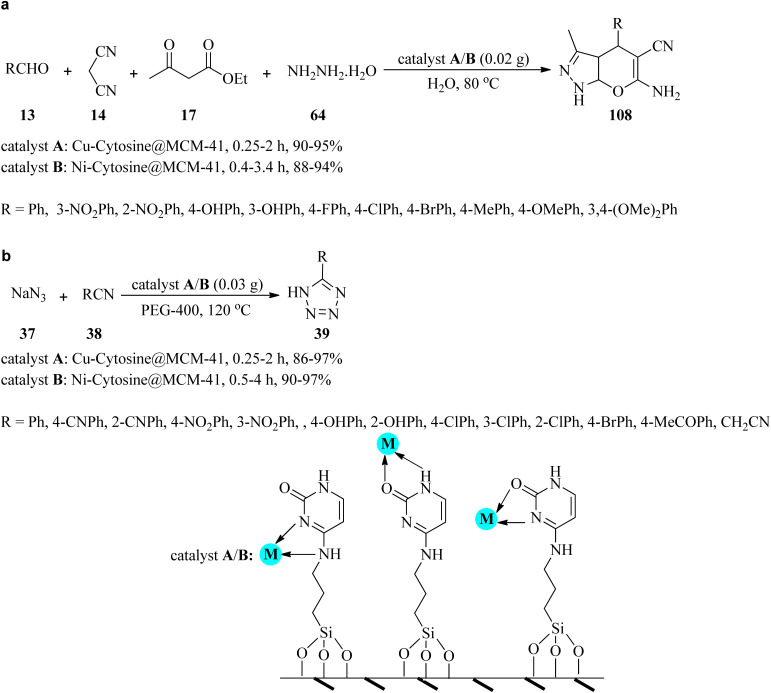
Synthetic approach for (a) pyranopyrazoles and (b) 5-substituted-1*H*-tetrazoles.

Due to the significant biological and pharmacological activities of pyranopyrazoles and tetrazoles,^136–138^ in 2020, Nikoorazm *et al.* presented an efficient and facile method for the preparation of pyranopyrazoles (108) and 5-substituted-1*H*-tetrazoles (39) by two catalytic systems, which are copper or nickel complexes of guanine confined in the mesoporous silica (Cu-cytosine@MCM-41 and Ni-cytosine@MCM-41). Pyranopyrazoles (108) were produced through the four-component condensation of equimolar amounts of aldehydes (13), 14, 17 and hydrazine hydrate (64) in water at 80 °C (Scheme 33a). On the other hand, the [3 + 2] cycloaddition of sodium azide (37) with nitriles (38) was accomplished by heating in polyethylene glycol (PEG-400) at 120 °C to give 5-substituted-1*H*-tetrazoles (39) (Scheme 33b). Based on the resulting data, both electron-donating and electron-withdrawing substituents on aldehydes and benzonitriles showed no significant effect on the reaction yields and the TOF. It was found that in both reactions, Cu-cytosine@MCM-41 had a great influence in decreasing the reaction time. Furthermore, the mesoporous framework of these catalysts was affirmed using nitrogen adsorption–desorption isotherms. Short reaction times, excellent yields, recyclability, and reusability of the synthesized nanocatalyst are some of the advantages of this process.^139^

**Scheme 34:**
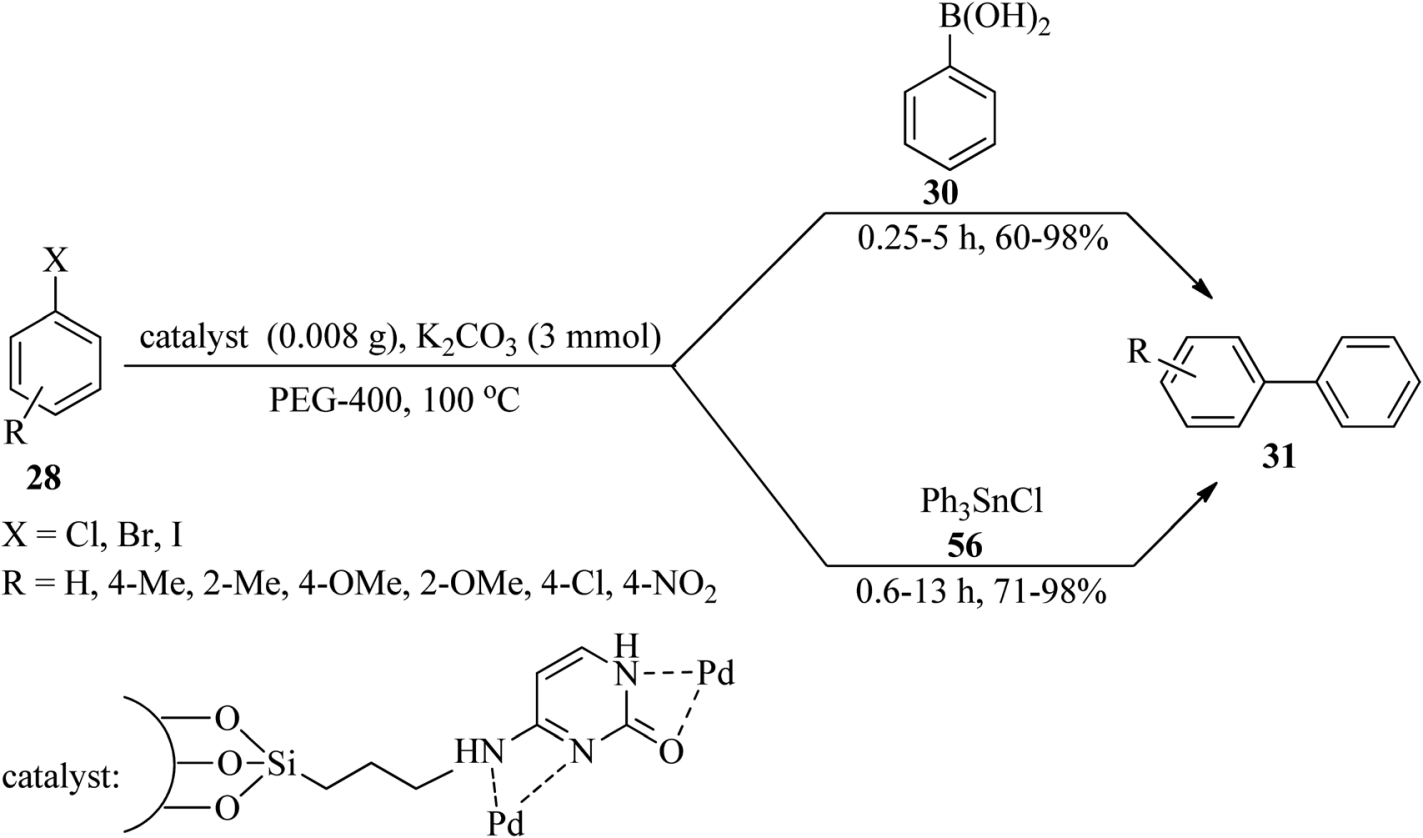
Biphenyl synthetic routes in the presence of HMS-CPTMS-Cy-Pd.

In 2019, Gholamian and Hajjami synthesized and characterized a Pd-cytosine complex stabilized on functionalized hexagonal mesoporous silica (HMS-CPTMS-Cy-Pd) as a novel, recyclable, and reusable catalyst. Its catalytic activity was investigated for the preparation of biphenyls *via* two pathways: (a) the Suzuki–Miyaura cross-coupling reaction of equimolar amounts of aryl halides (28) with phenylboronic acid (30) utilizing K_2_CO_3_ in PEG-400 at 100 °C gave rise to biphenyl compounds (31) in moderate to excellent yields (60–98%) within 0.25–5 h; (b) Stille reaction of aryl halides (28) and triphenyltin chloride (56), in a 1 : 0.5 molar ratio, under similar conditions yielded 31 (Scheme 34). The recyclability and reusability of the catalyst, experimental simplicity, short reaction times, and high yields are some of the advantages of this procedure.^140^

**Scheme 35:**
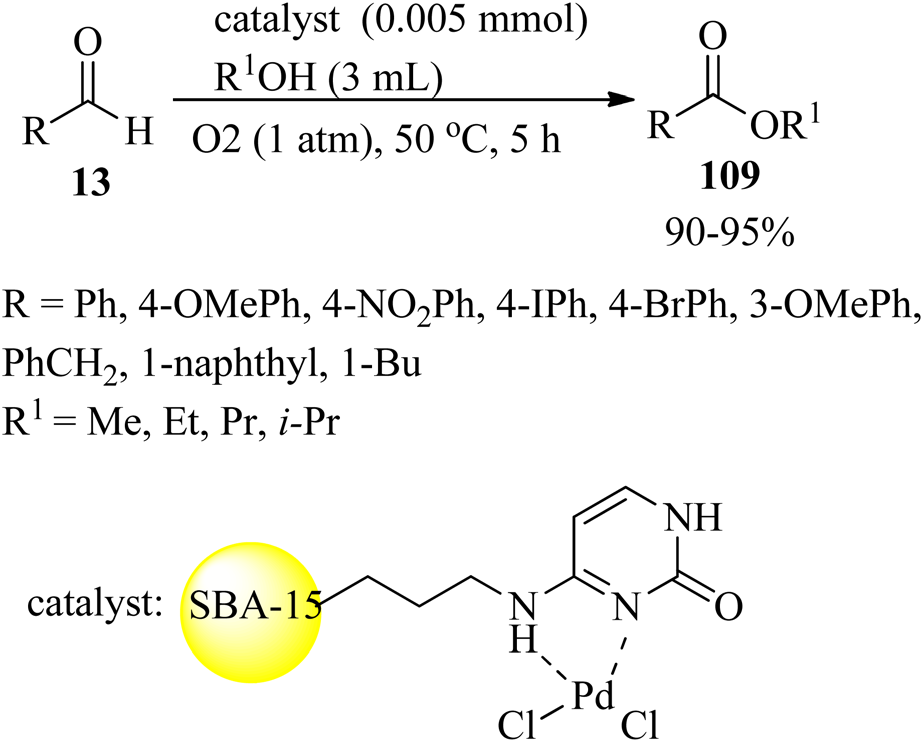
Oxidative esterification of aldehydes.

In 2021, Rajabi *et al.* constructed a cytosine palladium complex supported on ordered mesoporous silica (Pd-Cyt@SBA-15) that was utilized as a reusable nanocatalyst for the one-pot oxidative esterification of aldehydes (13). Diverse aliphatic, aromatic, and unsaturated aldehydes underwent oxidative transformation to the corresponding esters (109) in the presence of oxygen in large turnover numbers (Scheme 35). The Pd-Cyt@SBA-15 nanocatalyst demonstrated excellent reusability and stability up to ten times without loss of significant reactivity.^141^

Palladium on cytosine supported on SBA-15 nanoreactors (Pd-Cyt@SBA-15) also accelerated the formation of biphenyls (31) *via* the Suzuki–Miyaura reaction of various aryl chlorides (28) with boronic acid (30) in the presence of K_2_CO_3_ (1.5 mmol) base in a water/isopropanol (3 : 1) media at 60 °C in 88–98% yield within 6 h. Both electron-donating and electron-withdrawing aryl chlorides provided the corresponding products in excellent yields.^142^

In 2017, Rajabi *et al.* designed and identified novel highly-ordered periodic mesoporous silica cytosine functionalized nanomaterials (Cyt@SBA-15) for the synthesis of α,β-unsaturated dicyanides (55) through the Knoevenagel condensation of 14 with aldehydes (13)/ketones (74) in ethanol at room temperature in a 1 h period with 88–99% yields. It was found that the aldehydes bearing electron-withdrawing substituents gave the related products in excellent yields, which could probably be ascribed to the higher reactivity of their carbonyl moiety. The catalytic system also indicated a good performance for less reactive ketones, in comparison with aldehydes, to furnish the corresponding compounds in 88–92% yields. Mild reaction conditions, low catalyst loading, recyclability and reusability of the catalyst, high yields, and short reaction times are some of the main advantages of this strategy.^143^

## 5. Catalytic activity of thymine in two- and multi-component reactions

**Scheme 36:**
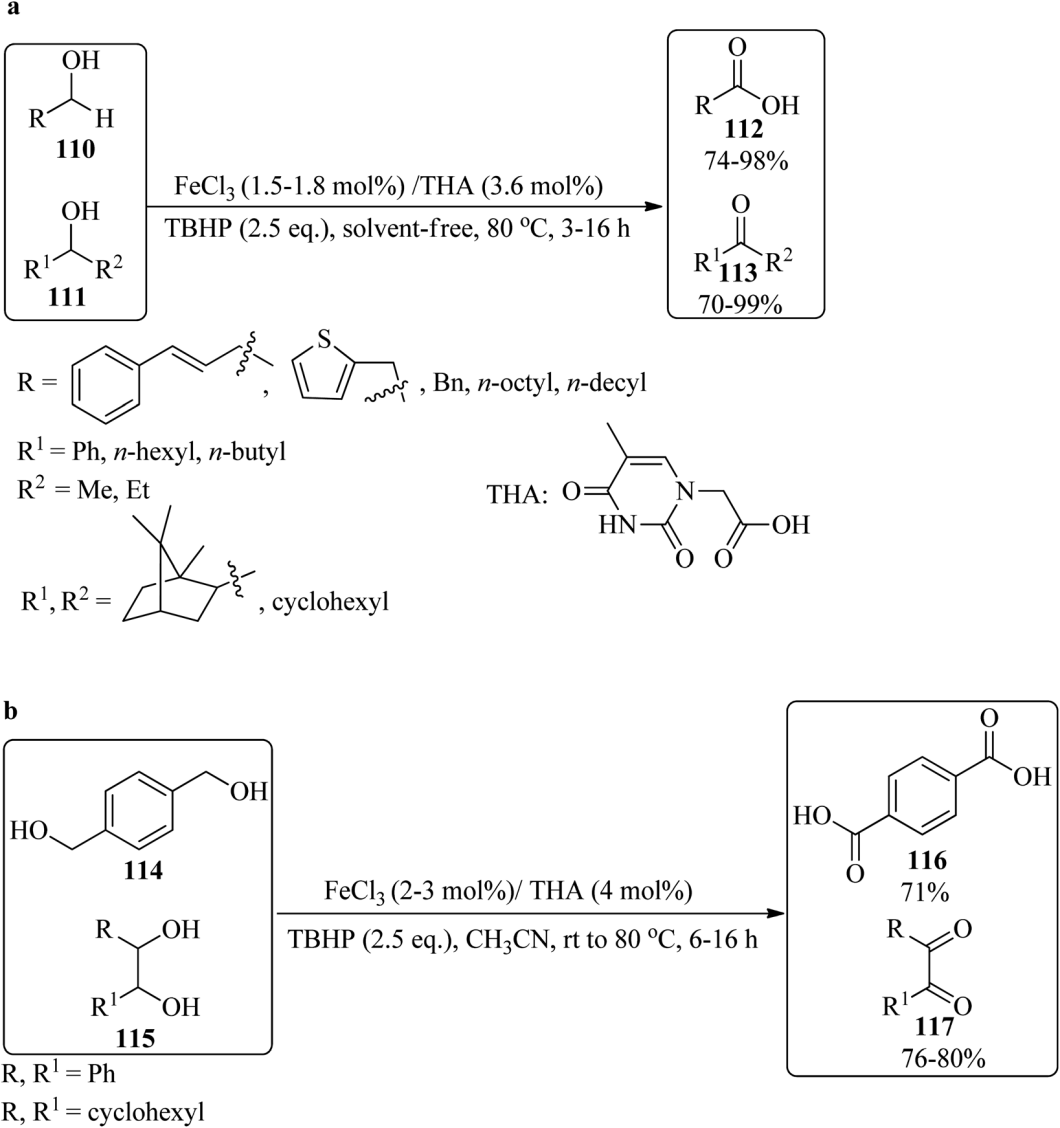
Oxidation of (a) primary and secondary alcohols, and (b) diols.

In 2010, Al-Hunaiti *et al.* produced thymine iron(iii) (THA/FeCl_3_) from the *in situ* reaction of anhydrous FeCl_3_ (1.8 mol%) with thymine-1-acetate (THA, 3.6 mol%). The oxidation reaction of diverse alcohols was performed under solvent-free conditions at 80 °C by TBHP as an oxidant. The reaction was performed on various primary/secondary alcohols (110/111), which were selectively oxidized to their corresponding carboxylic acids (112)/ketones (113), respectively (Scheme 36a). On the other hand, various diols, including 1,4-dibenzylic diol (114) and internal/cyclic *vic*-diols (115) (such as diphenyl ethanediol and 1,2-cyclohexanediol), were selectively transformed to phenyl dicarboxylic acid (116) and diketones (117), respectively (Scheme 36b). Mild reaction conditions, inexpensiveness, experimental simplicity, environmentally friendly, and high yields are advantages of this strategy.^144^

## 6. Conclusions

In this review, several types of nanocatalysts containing nucleobases have been presented. The sections have been classified according to the reactions catalyzed by each of the bases (A, G, C, T). Based on the overall results, the employment of nucleobases in the preparation of nanocatalysts, such as the metal-free and template-free *in situ* nitrogen-doped nanosheets, led to elevated catalytic performance, for instance, good stability (mechanical and/or thermal), high selectivity, high surface-to-volume ratio, clean energy production, eco-friendly, atom efficacy, time economic exploitation, and minimum chemical waste generation (green technology). Detailed examinations in the form of a literature survey revealed that due to the numerous interaction sites in adenine and guanine, and most organic reactions were carried out by multilayered nanocatalysts, including the immobilization of the mentioned nucleobases. The authors hope that this review article will guide general readers to attain an intense interest in utilizing the four main DNA nucleobases as promoters in various organic transformations. Due to the unique characteristics of these biochemical nucleobases, their activity in the field of green chemistry and medicinal/biochemical studies will definitely expand greatly in the future.

## Data availability

No primary research results, software or code have been included and no new data were generated or analysed as part of this review.

## Conflicts of interest

There are no conflicts to declare.
